# Loving-Kindness Meditation vs Cognitive Processing Therapy for Posttraumatic Stress Disorder Among Veterans: A Randomized Clinical Trial

**DOI:** 10.1001/jamanetworkopen.2021.6604

**Published:** 2021-04-16

**Authors:** David J. Kearney, Carol A. Malte, Meghan Storms, Tracy L. Simpson

**Affiliations:** 1 VA Puget Sound Health Care System, Seattle, Washington; 2 Division of Gastroenterology, Department of Medicine, University of Washington School of Medicine, Seattle; 3 Center of Excellence in Substance Addiction Treatment and Education, VA Puget Sound Health Care System, Seattle, Washington; 4 VA Research and Development, VA Puget Sound Health Care System, Seattle, Washington; 5 Department of Psychiatry and Behavioral Sciences, University of Washington School of Medicine, Seattle

## Abstract

**Importance:**

Additional options are needed for treatment of posttraumatic stress disorder (PTSD) among veterans.

**Objective:**

To determine whether group loving-kindness meditation is noninferior to group cognitive processing therapy for treatment of PTSD.

**Design, Setting, and Participants:**

This randomized clinical noninferiority trial assessed PTSD and depression at baseline, posttreatment, and 3- and 6-month follow-up. Veterans were recruited from September 24, 2014, to February 5, 2018, from a large Veternas Affairs medical center in Seattle, Washington. A total of 184 veteran volunteers who met *Diagnostic and Statistical Manual of Mental Disorders* (Fifth Edition) criteria for PTSD were randomized. Data collection was completed November 28, 2018, and data analyses were conducted from December 10, 2018, to November 5, 2019.

**Interventions:**

Each intervention comprised 12 weekly 90-minute group sessions. Loving-kindness meditation (n = 91) involves silent repetition of phrases intended to elicit feelings of kindness for oneself and others. Cognitive processing therapy (n = 93) combines cognitive restructuring with emotional processing of trauma-related content.

**Main Outcomes and Measures:**

Co–primary outcomes were change in PTSD and depression scores over 6-month follow-up, assessed by the Clinician-Administered PTSD Scale (CAPS-5; range, 0-80; higher is worse) and Patient-Reported Outcome Measurement Information System (PROMIS; reported as standardized T-score with mean [SD] of 50 [10] points; higher is worse) depression measures. Noninferiority margins were 5 points on the CAPS-5 and 4 points on the PROMIS depression measure.

**Results:**

Among the 184 veterans (mean [SD] age, 57.1 [13.1] years; 153 men [83.2%]; 107 White participants [58.2%]) included in the study, 91 (49.5%) were randomized to the loving-kindness group, and 93 (50.5%) were randomized to the cognitive processing group. The mean (SD) baseline CAPS-5 score was 35.5 (11.8) and mean (SD) PROMIS depression score was 60.9 (7.9). A total of 121 veterans (66%) completed 6-month follow-up. At 6 months posttreatment, mean CAPS-5 scores were 28.02 (95% CI, 24.72-31.32) for cognitive processing therapy and 25.92 (95% CI, 22.62-29.23) for loving-kindness meditation (difference, 2.09; 95% CI, −2.59 to 6.78), and mean PROMIS depression scores were 61.22 (95% CI, 59.21-63.23) for cognitive processing therapy and 58.88 (95% CI, 56.86-60.91) for loving-kindness meditation (difference, 2.34; 95% CI, −0.52 to 5.19). In superiority analyses, there were no significant between-group differences in CAPS-5 scores, whereas for PROMIS depression scores, greater reductions were found for loving-kindness meditation vs cognitive processing therapy (for patients attending ≥6 visits, ≥4-point improvement was noted in 24 [39.3%] veterans receiving loving-kindness meditation vs 9 (18.0%) receiving cognitive processing therapy; *P* = .03).

**Conclusions and Relevance:**

Among veterans with PTSD, loving-kindness meditation resulted in reductions in PTSD symptoms that were noninferior to group cognitive processing therapy. For both interventions, the magnitude of improvement in PTSD symptoms was modest. Change over time in depressive symptoms was greater for loving-kindness meditation than for cognitive processing therapy.

**Trial Registration:**

Clinicaltrials.gov Identifier: NCT01962714

## Key Points

**Question:**

Is group loving-kindness meditation noninferior to group cognitive processing therapy for treatment of posttraumatic stress disorder (PTSD) among veterans?

**Findings:**

In this randomized clinical trial, 184 veterans with PTSD were assigned to group loving-kindness meditation or group cognitive processing therapy; the differences in the decrease from baseline to 6-month follow-up for measures of PTSD and depression were very similar and within predefined margins considered not meaningfully different. Attendance was better for loving-kindness meditation.

**Meaning:**

This study adds to the evidence indicating that interventions without a specific focus on trauma, including meditation-based interventions, can yield results similar to trauma-focused therapies.

## Teaser

This randomized clinical trial assesses whether group loving-kindness meditation therapy yields a similar result to group cognitive processing therapy for the treatment of posttraumatic stress disorder among veterans treated at a VA medical center.

## Introduction

Military veterans are at increased risk of posttraumatic stress disorder (PTSD) and depression due to combat^1^ and other traumas.^2^ PTSD occurs in 15% to 26% of veterans deployed to Iraq or Afghanistan,^3,4^ 15% of male Vietnam veterans,^5^ and 2% to 12% of Gulf War I veterans.^6^ PTSD treatment guidelines recommend trauma-focused therapies, including cognitive processing therapy (CPT) and prolonged exposure, as first-line treatments.^7^ Despite the proven efficacy and successful dissemination of CPT and prolonged exposure in Veterans Affairs (VA) health care facilities, only half or fewer of veterans with PTSD enrolled in the VA seek care,^8,9^ and most who engage in treatment receive an inadequate amount of care.^9,10,11^ Barriers to PTSD treatment include stigma and concerns that medications will be required or that talking about trauma will be too difficult.^12^ New treatments tailored to patient preferences are necessary to achieve improved outcomes.^13,14^

Non–trauma-focused treatments can reduce PTSD symptoms.^15,16,17,18,19,20,21,22^ These interventions do not elicit trauma-related content but instead teach skills, such as mindfulness or problem-solving, which can be applied to situations in daily life.^16,23^ A non–trauma-focused intervention with preliminary support for treating PTSD^24,25^ is loving-kindness meditation, a practice intended to increase feelings of kindness and compassion. Loving-kindness meditation is theorized to increase the ability to tolerate, rather than avoid, distressing thoughts, images, and feelings and to counteract shame, guilt, and emotional numbing, which are central symptoms of PTSD^24,25^ (eAppendix in Supplement 2). This study tested whether loving-kindness meditation was noninferior to CPT. We hypothesized that veterans randomized to loving-kindness meditation would show reductions in PTSD and depression symptoms not meaningfully worse than those assigned to CPT. We also evaluated whether the proportion of veterans with clinically meaningful change, loss of PTSD diagnostic status, or attendance rates differed between the 2 treatments.

## Methods

### Design

This clinical trial, conducted from September 24, 2014, to February 5, 2018, compared veterans randomly assigned to group-based loving-kindness meditation or group-based CPT-C (cognitive only) on PTSD and depression outcomes at a large VA medical center serving the Pacific Northwest. The study was approved by the local institutional review board. All veterans provided written informed consent. This study followed the Consolidated Standards of Reporting Trials (CONSORT) reporting guideline.

### Participants

Demographic characteristics are shown in Table 1. To better describe the sample, veterans self-reported race/ethnicity using predefined categories or could choose “other.” Veterans met *Diagnostic and Statistical Manual of Mental Disorders* (Fifth Edition) (*DSM-5*) criteria for PTSD^26^ and agreed to not participate in mindfulness-based interventions, CPT, or prolonged exposure during the study. Exclusions were (1) substance use dependence disorder other than alcohol, (2) alcohol use that posed a safety concern, (3) high risk of suicide, (4) severe psychiatric illness (Supplement 1), or (5) prior participation in loving-kindness meditation or CPT. Medication and supportive counseling were allowed throughout the study.

**Table 1.** Demographic Characteristics of the Intention-to-Treat Sample

Characteristic	No. (%)^a^	
	CPT (n = 93)	LKM (n = 91)
Age, mean (SD), y	56.1 (13.7)	58.2 (12.5)
Sex		
Female	17 (18.3)	13 (14.3)
Male	76 (81.7)	77 (84.6)
Transgender	0	1 (1.1)
Race		
Black	20 (21.7)	24 (26.4)
Asian or Pacific Islander	3 (3.3)	3 (3.3)
American Indian	3 (3.3)	0
White	54 (58.7)	53 (58.2)
Other or multiple	12 (13.0)	11 (12.1)
Ethnicity Hispanic or Latino	6 (6.4)	3 (3.3)
Employment		
Employed	21 (23.3)	17 (18.7)
Unemployed	5 (5.6)	8 (8.8)
Unemployed due to disability	31 (34.4)	29 (31.9)
Retired	24 (26.7)	33 (36.3)
Student/homemaker	9 (10)	4 (4.4)
Education		
High school or less	12 (12.9)	12 (13.2)
Some college	36 (38.7)	45 (49.4)
College degree	45 (48.4)	34 (37.4)
Service-connected disability ≥50%	67 (72.0)	57 (62.6)
Prior mental health or SUD inpatient admission	34 (37.4)	34 (39.1)
Psychotropic medication use at baseline		
Antidepressants	51 (54.8)	53 (58.2)
Benzodiazepines	16 (17.2)	10 (11.0)
Other antianxiety medications	13 (14.0)	7 (7.7)
Antipsychotics	16 (17.2)	9 (9.9)
Mood stabilizers	3 (3.2)	2 (2.2)
Type of trauma event		
Combat	50 (53.8)	47 (51.6)
Sexual assault or unwanted sexual contact	18 (19.4)	15 (16.5)
Other assault	8 (8.6)	12 (13.2)
Accident	7 (7.5)	5 (5.5)
Sudden death	6 (6.4)	8 (8.8)
Other	4 (4.3)	4 (4.4)
Baseline CAPS-5 total score, mean (SD)	35.5 (11.5)	35.5 (12.1)
Baseline PROMIS depression T-score, mean (SD)	60.5 (7.6)	61.3 (8.2)
Treatment session attendance		
Total study treatment sessions, mean (SD)^b^	6.0 (4.6)	7.4 (4.2)
Attended ≥6 treatment sessions	50 (53.8)	61 (67.0)
Other mental health visits (baseline to 6-mo follow-up), mean (SD)	6.4 (9.3)	7.1 (10.2)

Abbreviations: CAPS-5, Clinician-Administered PTSD Scale; CPT, cognitive processing therapy; PROMIS, Patient-Reported Outcomes Measurement Information System; PTSD, posttraumatic stress disorder; SUD, substance use disorder.

^a^ Values are expressed as No. (%) unless otherwise specified.

^b^ *P* < .05.

### Procedures

**Figure 1.**
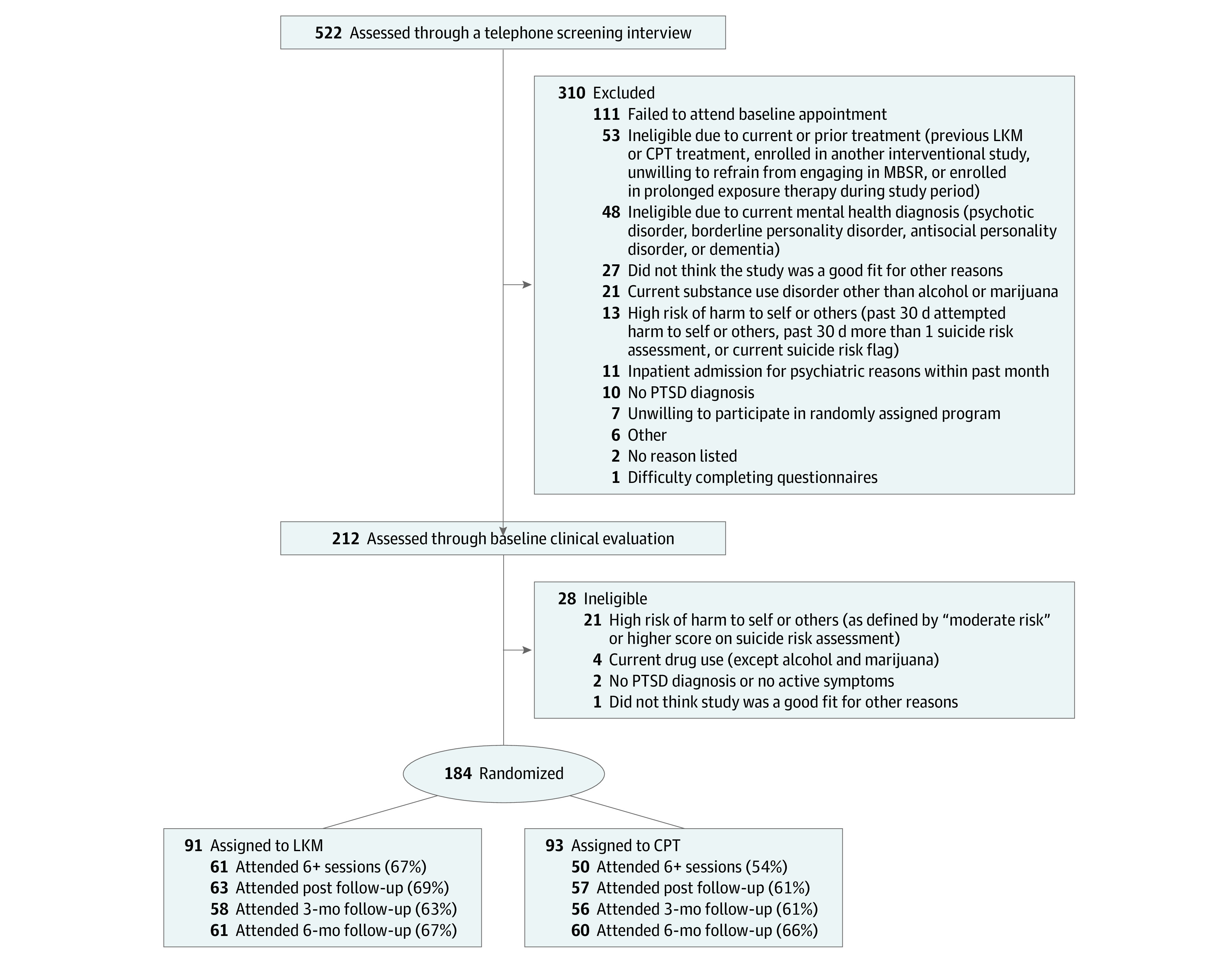
Consolidated Standards of Reporting Trials (CONSORT) Flow Diagram CPT indicates cognitive processing therapy; LKM, loving-kindness meditation; MBSR, mindfulness-based stress reduction.

Recruitment consisted primarily of mailings to veterans with a diagnosis of PTSD who received VA care, as identified via VA databases. A telephone screen was followed by a 2-hour in-person baseline assessment. Twelve cohorts of 10 to 21 veterans were randomized every 3 to 4 months from September 24, 2014, to February 5, 2018. Figure 1 illustrates recruitment and retention. Participants completed assessments at posttreatment and 3-month and 6-month follow-ups and received $20 to $50 for each assessment.

#### Randomization

Random permuted block randomization (blocks of 2, 4, or 6 individuals) stratified by symptom severity per the Clinician-Administered PTSD Scale (CAPS-5, score ≥37) with masked allocation using sequentially numbered opaque envelopes was performed. The CAPS-5 score was calculated by summing severity scores for the 20 *DSM-5* PTSD symptoms. A symptom was considered present if the severity was 2 (moderate) or higher. A PTSD diagnosis required at least 1 symptom for both criteria B and C, at least 2 symptoms for both criteria D and E, and meeting criteria F and G. A staff member not involved in recruitment constructed randomization lists using the random number generator feature of Excel (Microsoft Corp).^27^

#### Study Therapists

Loving-kindness meditation groups were co-led by 2 community meditation teachers with experience teaching mindfulness and loving-kindness meditation to veterans. A study team member (D.J.K.) with experience teaching loving-kindness meditation provided supervision. CPT groups were co-led by 2 master’s-level therapists who had completed training and certification in CPT through the VA. They received supervision by an experienced CPT national trainer.

### Treatment Conditions

Each intervention consisted of 12 weekly 90-minute group sessions including male and female veterans. The study design provided parity in number of treatment hours, treatment sessions, and the number and allegiance of facilitators and therapists across conditions.

### Loving-Kindness Meditation

The loving-kindness meditation curriculum is based on the formulation described by Salzberg.^28^ In loving-kindness meditation, one calls to mind a particular person (eg, a good friend) and silently repeats phrases that invoke goodwill, such as “may you be safe,” “may you be happy,” and “may you be healthy.” The practice gradually expands to include oneself and those who have caused difficulty or harm.^28^ Veterans were asked to notice thoughts and feelings elicited by the phrases with an attitude of kindness, curiosity, and nonjudgment, regardless of content. Class sessions began with a brief mindfulness (weeks 1 and 2) or loving-kindness (weeks 3 through 12) meditation followed by discussion and additional loving-kindness meditation practice. Educational materials describing the relationship between meditation, PTSD, and depression were provided. Homework consisted of 30 minutes of meditation 6 days per week using compact discs and informal loving-kindness meditation practices in daily life.

### CPT (Cognitive Only)

CPT-C is based on Resick and colleagues’ manual for treating PTSD among military veterans, which combines cognitive restructuring with emotional processing of trauma-related content^29^ but does not include writing a trauma narrative.^30^ Sessions initially focus on rigid or inaccurate beliefs about the traumatic event, which often reflect self-blame or hindsight bias. Later sessions address trauma-related, overgeneralized beliefs about self and others relevant to 5 key areas: safety, trust, power, esteem, and intimacy. Participants learn to identify and modify their beliefs to become more balanced, flexible, and adaptive. Homework, conducted for 30 minutes 6 days per week, consisted of completing worksheets and exercises as well as writing an impact statement at the beginning and end of treatment.

### Measures

PTSD severity and diagnostic status were assessed using the 30-item CAPS-5 (score range, 0-80; higher scores indicate more severe PTSD) structured interview^31^ with lifetime number and type of traumatic events assessed via the 17-item Life Events Checklist.^32^ The CAPS-5 has strong psychometric properties.^31^ A clinician (M.S.) masked to the randomization arm conducted the interviews. Depression was assessed using the Patient-Reported Outcomes Measurement Information System (PROMIS) depression measure (PROMIS scores are reported as a standardized T-score, which represents a mean [SD] of 50 [10] points; higher scores indicate worse depression),^33^ which has high internal consistency (Cronbach α = 0.979)^34^ and precision. Other VA mental health care received by participants between baseline and 6-month follow-up was extracted from VA databases, and days of mental health treatment received for each treatment arm were calculated.

### Outcomes

The primary outcome of noninferiority of loving-kindness meditation relative to CPT-C was assessed using a noninferiority margin of 5 points on the CAPS-5, which represents 0.5 SD of baseline PTSD symptoms based on data from a large sample (N = 198) of treatment-seeking veterans (B.P. Marx, MD, email communication, 2019). An effect size of Cohen *d* = 0.5 has been defined as the minimally important difference in prior PTSD trials.^35,36,37^ The proportion of participants with clinically meaningful improvement or worsening of PTSD symptoms was assessed using both stringent (≥10 points) and less stringent (≥5 points) criteria. Loss of PTSD diagnostic status was calculated as the proportion no longer meeting *DSM-5* criteria,^26^ and full remission was calculated as the proportion with a CAPS-5 score less than 12.^38^ For depression, the noninferiority margin was 4 points on the PROMIS depression measure, which is defined as the minimally important difference and represents a Cohen *d* effect size of approximately 0.50.^39,40,41^ The proportion with clinically meaningful improvement or worsening of depressive symptoms was assessed using stringent (≥8 points) and less stringent (≥4 points) criteria.

### Treatment Fidelity and Adverse Events

All sessions were recorded, and a random subset (20%) was coded for adherence, competence, and proscribed elements by 2 independent raters. Training on loving-kindness meditation fidelity coding was provided by a study team member (D.J.K.), and training on CPT-C fidelity coding was provided by a psychologist with extensive experience coding CPT. For loving-kindness meditation, 92.8% of essential elements were delivered, and there were no proscribed elements. Competence in loving-kindness meditation delivery was rated on a 7-point scale (7 = excellent, 4 = satisfactory), with a mean (SD) competence score of 5.4 (0.5) or “good”; 96.3% of the elements were rated “satisfactory” or better. For CPT, 98.4% of unique and essential elements were included in all sessions, and there were no proscribed elements. Competence of CPT delivery was rated on a 5-point scale (5 = excellent, 3 = satisfactory); the mean (SD) therapist competence score was 4.0 (0.5) or “good,” and 97% of all elements were rated “satisfactory” or better. Adverse events were prospectively monitored, including hospitalizations, suicidality, death, and a prespecified level of increase in PTSD or depression score.

### Statistical Analysis

Data collection was completed November 28, 2018, and data analyses were conducted from December 10, 2018, to November 5, 2019. Sample size was determined using Stata, version 15 (StataCorp LLC) using the SSI module for noninferiority trials.^42^ For CAPS-5 severity scores, a noninferiority margin of 5 points, SD of 10, a 1-sided α of 0.025,^36^ power of 0.80, and equal allocation of participants between treatments indicated that 63 patients per randomization arm were needed. To protect against an anticipated attrition rate of 26%, the sample was inflated to 170 patients. Characteristics of veterans who completed half or more of the treatment sessions were compared with those who did not using χ^2^ analysis and *t* tests for categorical and continuous data, respectively.

Noninferiority was assessed using linear mixed models that included treatment condition, time (baseline, posttreatment, 3 months, and 6 months), and time by treatment interaction as fixed effects. Because outcomes were highly correlated for both the loving-kindness meditation (intraclass correlation coefficient [ICC], 0.55; 95% CI, 0.41-0.68) and CPT-C (ICC, 0.58; 95% CI, 0.46-0.70) at the individual level, a patient identifier was included as a random effect to account for correlated outcomes over time. Correlation at the group cohort level was found to be minimal and not included in the final models. Age, sex, trauma type, and baseline PROMIS depression and CAPS-5 scores for the PTSD and depression noninferiority models, respectively, were included as covariates. A patient identifier was included as a random effect to account for correlated outcomes over time (ICC, 0.44; 95% CI, 0.22-0.68 and 0.45; 95% CI, 0.28-0.62 in the loving-kindness meditation and CPT-C arms, respectively). The noninferiority of loving-kindness meditation with respect to CPT-C was analyzed using the 95% CI for the group × time interaction term, with noninferiority of loving-kindness meditation to CPT-C claimed if the lower limit of the 95% CI was greater than –δ. Analyses were performed using both intention-to-treat (ITT) and completer (those attending ≥6 sessions of loving-kindness meditation or CPT-C) samples given that ITT analyses may bias results toward noninferiority,^43^ with noninferiority claimed if both completer and ITT analyses demonstrated noninferiority^36,43^ at the 6-month time point. If noninferiority was shown, as part of the analytic plan for the primary aim, we assessed superiority using the group × time interaction term from the linear mixed models described previously, with a 2-sided α of .05 considered significant.^44,45^

Between- and within-group effect sizes based on the previously mentioned models were calculated as Cohen *d*. Proportions with clinically meaningful change, full remission, and no longer meeting *DSM-5* criteria were compared using χ^2^ tests. Veterans with missing data were coded as not demonstrating clinically meaningful change and as retaining diagnostic status. All analyses were completed in Stata, version 15 (StataCorp LLC).

## Results

Among the 184 veterans (mean [SD] age, 57.1 [13.1] years; 153 men [83.2%]; 107 White participants [58.2%]) included in the study, 91 (49.5%) were randomized to the loving-kindness group, and 93 (50.5%) were randomized to the cognitive processing group. PTSD and depression symptom severity were similar at baseline across the 2 conditions (Table 1). The mean number of treatment sessions completed was lower in CPT than in loving-kindness meditation (mean [SD] sessions, 6.01 [4.6] vs 7.40 [4.2], respectively; *P* = .03). Veterans who completed 6 or more sessions (n = 111) were older (≥6 sessions, mean [SD] age, 59.4 [12.3] years; <6 sessions, 53.7 [13.7] years; *P* = .004) and had greater baseline depression (≥6 sessions, mean [SD], 69.6 [7.3]; <6 sessions, 62.9 [8.3]; *P* = .005).

**Figure 2.**
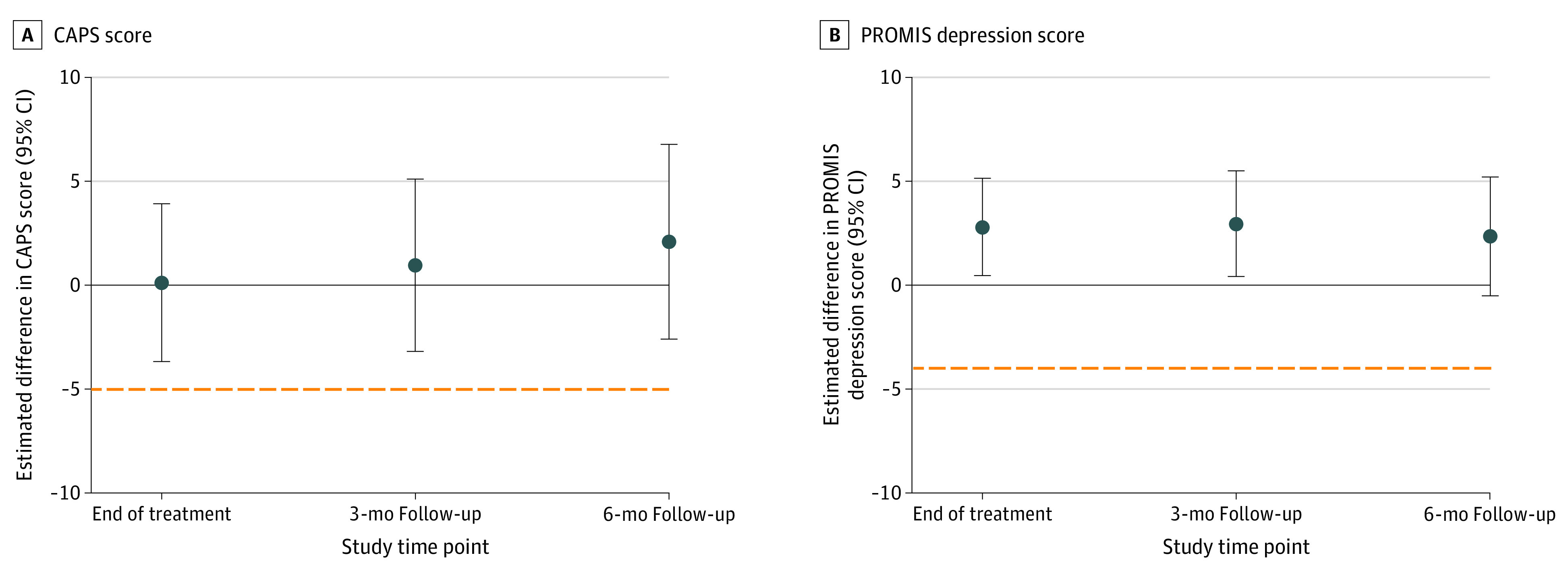
Estimated Difference Between Clinician-Administered Posttraumatic Stress Disorder Scale (CAPS) and Patient-Reported Outcome Measurement Information System (PROMIS) Depression Score at the End of Treatment and During Follow-up Analyzing the difference between (A) CAPS and (B) PROMIS scores, for loving-kindness meditation (LKM) to be noninferior to cognitive processing therapy (CPT), the lower bound of the 2-sided 95% CI for the difference in change between treatments (CPT – LKM) must be greater than the noninferiority margin (5 for CAPS, 4 for PROMIS depression) and is indicated by the orange dotted line. Circles show the mean difference between CPT and LKM; error bars indicate 2-sided 95% CIs around the means.

Table 2 shows estimated means over time for CAPS-5 and PROMIS depression scores. Differences in mean scores at each time point for primary outcomes in relation to the noninferiority margin are illustrated in Figure 2 and the eFigure in Supplement 2. The mean (SD) baseline CAPS-5 score was 35.5 (11.8), and the mean (SD) PROMIS depression score was 60.9 (7.9). A total of 121 (66%) veterans completed 6-month follow-up. At 6 months posttreatment, mean CAPS-5 scores were 28.02 (95% CI, 24.72-31.32) for CPT and 25.92 (95% CI, 22.62-29.23) for loving-kindness meditation (difference, 2.09; 95% CI, −2.59 to 6.78), and mean PROMIS depression scores were 61.22 (95% CI, 59.21-63.23) for CPT and 58.88 (95% CI, 56.86-60.91) for loving-kindness meditation (difference, 2.34; 95% CI, −0.52 to 5.19). In completer models, the differences in decrease were 1.94 (95% CI, −3.70 to 7.59) for CAPS-5 scores and 3.52 (95% CI, 0.39 to 6.66) for PROMIS depression scores. The lower bound of the 95% CI of the difference between treatments was greater than the noninferiority margin for both CAPS-5 and PROMIS depression in ITT and completer analyses at 6-month follow-up, indicating that the comparisons met criteria for noninferiority. In superiority analyses, for CAPS-5 score, the group × time interaction term was not significant at any time point in both ITT and completer analyses. For PROMIS depression scores, in ITT analyses, the group × time interaction was significant at the posttreatment (β = 3.63; 95% CI, 1.23-6.04 points; *P* = .003), 3-month (β = 3.79; 95% CI, 1.22-6.36 points; *P* = .004), and 6-month (β = 3.17; 95% CI, 0.31-6.04 points; *P* = .03) visits, indicating superiority of loving-kindness meditation to CPT. Effect sizes ranged from 0.35 at posttreatment to 0.24 at 6 months, indicating a small effect. Completer analyses showed similar results with a significant group × time interaction at posttreatment (β = 3.79; 95% CI, 1.10-6.48 points; *P* = .006), 3-month (β = 3.21; 95% CI, 0.39-6.03 points; *P* = .03), and 6-month (β = 3.66; 95% CI, 0.70-6.62; *P* = .02) visits. Effect sizes were slightly larger, ranging from 0.54 at posttreatment to 0.42 at 6 months, indicating a small to medium effect. These findings indicate greater improvement in depression symptoms but not PTSD symptoms from baseline to follow-up for loving-kindness meditation relative to CPT.

**Table 2.** Estimated Outcome Measures by Condition and Time Point^a^

Outcome measure	Patient No.	Estimated mean (95% CI)			
		Baseline	End of treatment	3-mo follow-up	6-mo follow-up
**ITT**					
CAPS-5 score^b^	183				
CPT	93	35.18 (32.94-37.42)	29.47 (26.77-32.17)	29.09 (26.16-32.01)	28.02 (24.72-31.32)
LKM	90	34.99 (32.72-37.26)	29.35 (26.70-31.99)	28.12 (25.20-31.04)	25.92 (22.62-29.23)
PROMIS depression T-score^c^	184				
CPT	93	60.43 (59.08-61.79)	61.25 (59.58-62.93)	62.06 (60.27-63.86)	61.22 (59.21-63.23)
LKM	91	61.27 (59.89-62.64)	58.46 (56.84-60.07)	59.1 (57.33-60.88)	58.88 (56.86-60.91)
**Participants completing ≥6 treatment sessions**					
CAPS-5 score	110				
CPT	50	33.73 (30.67-36.78)	27.53 (24.27-30.79)	27.50 (23.96-31.04)	27.70 (23.63-31.77)
LKM	60	34.36 (31.58-37.14)	29.20 (26.19-32.20)	28.30 (24.94-31.65)	25.76 (21.92-29.60)
PROMIS depression T-score^c^	111				
CPT	50	59.60 (57.88-61.32)	60.79 (58.93-62.66)	61.16 (59.13-63.19)	60.46 (58.21-62.71)
LKM	61	59.74 (58.18-61.30)	57.14 (55.44-58.84)	58.09 (56.17-60.01)	56.93 (54.79-59.08)

Abbreviations: CAPS-5, Clinician-Administered PTSD Scale; CPT, cognitive processing therapy; ITT, intention to treat; LKM, loving-kindness meditation; PROMIS, Patient-Reported Outcomes Measurement Information System; PTSD, posttraumatic stress disorder.

^a^ Data from 1 veteran are missing from the PTSD models because the subject did not complete the PROMIS depression at baseline, which is a covariate.

^b^ Adjusted for sex, age, baseline PROMIS depression T-score, and trauma type.

^c^ Adjusted for sex, age, baseline CAPS-5 score, and trauma type.

The CAPS-5 within-condition effect sizes at 6 months were medium for CAPS-5 for loving-kindness meditation (*d* = 0.66; 95% CI, 0.36-0.96) and CPT (*d* = 0.52; 95% CI, 0.22-0.81) in ITT analyses, whereas between-condition effect sizes included 0 (*d* = 0.13; 95% CI, –0.16 to 0.42). Similar findings were seen in CAPS-5 completer analyses. For PROMIS depression, within-condition effect sizes for both loving-kindness meditation (*d* = 0.28; 95% CI, –0.01 to 0.58) and CPT (*d* = 0.09; 95% CI, –0.38 to 0.19) included 0 at 6 months in ITT analyses, as did between-condition effect sizes (*d* = 0.24; 95% CI, –0.05 to 0.53). For PROMIS depression completer analyses, within-condition effect sizes at 6 months were small to medium for loving-kindness meditation (*d* = 0.38; 95% CI, 0.02-0.73) and included 0 for CPT (*d* = –0.12; 95% CI, –0.51 to 0.27). Between-condition effect sizes demonstrated a medium effect favoring loving-kindness meditation (*d* = 0.42; 95% CI, 0.04-0.80). Effect sizes are summarized in the eTable in Supplement 2.

The proportions with clinically meaningful improvement or loss of diagnostic status are presented in Table 3. For CAPS-5, no differences by treatment arm were found for these parameters in both ITT and completer samples. For PROMIS depression, greater reductions were found for loving-kindness meditation vs CPT (for patients attending ≥6 visits, ≥4-point improvement was noted in 24 [39.3%] veterans receiving loving-kindness meditation vs 9 [18.0%] receiving CPT; *P* = .03), with no differences seen at other time points or using more stringent criteria (improvement ≥8 points). Results were similar in the completer sample.

**Table 3.** Improvement Over Time by Condition^a^

Condition	No. (%)					
	Posttreatment		3-mo follow-up		6-mo follow-up	
	CPT	LKM	CPT	LKM	CPT	LKM
**ITT**						
CAPS-5 improvement						
≥10 Points	18 (19.4)	23 (25.3)	17 (18.3)	20 (22.0)	23 (24.7)	25 (27.5)
≥5 Points	27 (29.0)	33 (36.3)	31 (33.3)	36 (39.6)	35 (37.6)	38 (41.8)
Loss of DSM-5 diagnosis per CAPS-5	27 (29.0)	25 (27.5)	23 (24.7)	28 (30.8)	30 (32.3)	36 (39.6)
PTSD remission (CAPS-5 <12)	6 (6.5)	9 (9.9)	6 (6.5)	6 (6.6)	10 (10.8)	10 (11.0)
PROMIS depression T-score improvement						
≥8 Points	4 (4.3)	11 (12.1)	6 (6.5)	14 (15.4)	7 (7.5)	11 (12.1)
≥4 Points	13 (14.0)	28 (30.8)^b^	14 (15.1)	22 (24.2)	16 (17.2)	25 (27.5)
**Completers: patients attending ≥6 visits**						
CAPS-5 improvement						
≥10 Points	15 (30.0)	19 (31.2)	15 (30.0)	16 (26.2)	16 (32.0)	19 (31.2)
≥5 Points	23 (46.0)	28 (45.9)	25 (50.0)	28 (45.9)	23 (46.0)	29 (47.5)
Loss of DSM-5 diagnosis per CAPS-5	25 (50.0)	22 (36.1)	20 (40.0)	21 (34.4)	22 (44.0)	27 (44.3)
PTSD remission (CAPS-5 <12)	5 (10.0)	7 (11.5)	6 (12.0)	4 (6.6)	8 (16.0)	8 (13.1)
PROMIS depression T-score improvement						
≥8 Points	3 (6.0)	10 (16.4)	4 (8.0)	11 (18.0)	4 (8.0)	10 (16.4)
≥4 Points	9 (18.0)	24 (39.3)^c^	11 (22.0)	17 (27.9)	11 (22.0)	21 (34.4)

Abbreviations: CAPS-5, Clinician-Administered PTSD Scale; CPT, cognitive processing therapy; DSM-5, *Diagnostic and Statistical Manual of Mental Disorders* (Fifth Edition); ITT, intention to treat; PROMIS, Patient-Reported Outcomes Measurement Information System; PTSD, posttraumatic stress disorder.

^a^ Veterans with missing data coded as not improved.

^b^ *P* < .01.

^c^ *P* < .05.

### Adverse Events

Serious adverse events included 1 suicide attempt in the CPT arm and 2 deaths (due to cancer) and 1 inpatient psychiatric admission (bipolar mania) in the loving-kindness meditation arm. No serious adverse events were deemed study related. Other adverse events included 1 disenrollment due to risk of harm to others, 4 incidents of suicidality with intent or plan in the CPT arm, and 1 seizure in the loving-kindness meditation arm. There were no significant differences between treatment arms in the severity or frequency of adverse events.

The number of veterans with at least a 20-point increase in CAPS-5 PTSD score or an increase of 2 categories in depression severity (using PROMIS scores transformed to Patient Health Questionnaire-9 scores)^34^ between assessments were 3 for PTSD and 15 for depression in the CPT group and 4 for PTSD and 7 for depression in the loving-kindness meditation group.

## Discussion

This study supports the use of loving-kindness meditation as a treatment for PTSD among veterans. Loving-kindness meditation was noninferior to CPT for PTSD and depressive symptoms at 6-month follow-up, although the magnitude of improvement for both interventions was modest. Change over time in PTSD symptoms was not found to be superior for either loving-kindness meditation or CPT at any time point, but change over time in depressive symptoms was superior for loving-kindness meditation compared with CPT at all time points. Those randomized to loving-kindness meditation also attended significantly more treatment sessions, suggesting that loving-kindness meditation was as acceptable as CPT to veterans with PTSD among our recruited sample; however, only 54% and 60% of participants attended 6 or more sessions of CPT or loving-kindness meditation, respectively.

Our results are consistent with other studies indicating that interventions without a specific focus on trauma-related symptomatology can yield results similar to trauma-focused interventions.^15,16,17,18,19,20,21,22,46^ The low completion rates for both interventions is consistent with completion rates ranging from 39% to 68% in other PTSD studies involving veterans.^47,48,49^ In the ITT sample, the proportions with clinically meaningful change (≥5 points on CAPS-5) and no longer meeting DSM-5 criteria for PTSD at 6 months are similar to those from a review of 5 randomized clinical trials of CPT for military-related PTSD, which found that 49% reported clinically meaningful change, and 28% to 40% no longer met criteria for PTSD.^17^

One explanation for the modest effects found for PTSD symptom reduction in the CPT cohort is the group delivery format. Individually delivered CPT results in greater reductions in PTSD symptoms than group CPT.^50^ Studies of active-duty military personnel undergoing group CPT found medium^50^ to large^51^ within-condition effect sizes. One trial assessed outcomes of group CPT for veterans with PTSD and at 6-month follow-up found that 26% no longer met criteria for PTSD and a within-condition effect size (Cohen *d*) of 0.76,^37^ consistent with our results.

### Strengths and Limitations

Strengths of the study include use of a conservative noninferiority margin of 5 points for the CAPS-5, which is narrower than the 10-point margin used by others.^52^ Other strengths are the study design (which accounted for elements of interventions known to contribute to change),^53^ assessment of treatment fidelity, masked clinician rating of PTSD outcomes, and 6-month follow-up.

Limitations of the study include a predominantly White male sample of veterans from 1 facility, which limits generalizability; and high rates of noncompletion of interventions, which could bias toward noninferiority. Statistical comparisons of secondary outcomes should be considered exploratory given lack of adjustment for α level. In addition, there were no measures of treatment credibility or the adequacy of masking of the assessor of PTSD symptoms.

## Conclusions

In this randomized clinical trial with a sample of veterans with PTSD, group loving-kindness meditation resulted in reductions in PTSD symptoms that were not meaningfully worse than group CPT and higher attendance relative to CPT. For both interventions, the magnitude of PTSD symptom reduction and rates of clinically meaningful improvement or loss of PTSD diagnostic status were similar to outcomes reported in prior studies of veterans with PTSD. Improvement over time in depressive symptoms was significantly greater for the loving-kindness meditation cohort relative to CPT, although few differences were detected in rates of clinically meaningful improvement. Further qualitative research would help to clarify the acceptability of loving-kindness meditation for PTSD. Overall, loving-kindness meditation shows promise as a treatment for PTSD, and the findings warrant replication.

## Notes

Supplement 1. **Trial Protocol**

Supplement 2. **eAppendix.** Additional Description of Loving-Kindness Meditation and Rationale for Its Use as a PTSD Treatment **eTable.** Within- and Between-Condition Effect Sizes by Treatment Outcome **eFigure.** Estimated Differences Between Loving-Kindness Meditation and Cognitive Processing Therapy in Outcome Measures by Time, Patients Attending ≥6 Visits **eReferences**

Supplement 3. **Data Sharing Statement**
