# Errors in Abstract and Discussion

**DOI:** 10.1001/jamanetworkopen.2021.15843

**Published:** 2021-05-27

In the Original Investigation titled “Loving-Kindness Meditation vs Cognitive Processing Therapy for Posttraumatic Stress Disorder Among Veterans: A Randomized Clinical Trial,”^1^ published April 16, 2021, the word “Veterans” was misspelled in the Abstract, and in the Results, the percentage 67% was incorrectly written as 60% in the sentence “Those randomized to loving-kindness meditation also attended significantly more treatment sessions, suggesting that loving-kindness meditation was as acceptable as CPT to veterans with PTSD among our recruited sample; however, only 54% and 67% of participants attended 6 or more sessions of CPT or loving-kindness meditation, respectively.” This article has been corrected.^1^
