# Supplementary material for: Loving-Kindness Meditation vs Cognitive Processing Therapy for Posttraumatic Stress Disorder Among Veterans: A Randomized Clinical Trial
Source: JAMA Netw Open. 2021 Apr 16;4(4):e216604. doi: 10.1001/jamanetworkopen.2021.6604 (PMC8052593; doi:10.1001/jamanetworkopen.2021.6604)
Supplement: Supplement 1. — Trial Protocol [file jamanetwopen-e216604-s001.pdf]

Study Protocol

Loving-Kindness Meditation and Cognitive Processing Therapy for Posttraumatic Stress Disorder Among Veterans: A Randomized Non-Inferiority Clinical Trial

Contents of this protocol summary:

- **Summary of protocol changes**
- **IRB-approved study protocol**

Table: Summary of Protocol Changes

Date	Description of Change
March 2014	Prior to initiation of study, refinement of measurement battery, including:<br>• Change in frequency of administration of of pain intensity measure, adding NIH PROMIS GI distress measure, adding curiosity subscale of Toronto Mindfulness Scale, changing FFMQ to 24-item version<br>• Refinement of safety monitoring questions to include ideation of harm to self or others
April 2014	Prior to initiation of study, refinement of measurement battery, including:<br>• Refinement of safety monitoring to include assessment of suicidal intent<br>• Replacement of interim cohort analyses of PTSD and depressive symptoms with individual subject monitoring for AEs<br>• Updating AEs to include: worsening of PTSD/depressive symptoms, worsening alcohol use), refined questions about alcohol consumption<br>• Removed exclusion criteria of prior MBSR participation and willingness to forego participation in yoga programs during study
June 2014	Revised data collection method for weekly measures: replaced Survey Monkey with paper assessments
August 2014	Revised phone screen:<br>• Removed question about presence of thought disorder; removed question about whether veterans is 18 years old, removed question about whether they have PTSD<br>Removed IBS-SSS from assessments and global IBS question, replaced with PROMIS GI symptom scales
October 2014	1. Added recruitment letter for veterans interested in study but cannot be reached by phone<br>2. Revised MINI Psychiatric Interview<br>a. Removed sections J and K<br>b. Replaced w/ AUCIT-C at baseline<br>c. Add DAST-10 at major assessments for marijuana use<br>d. Added drug cart sort at baseline<br>e. Added 1st 3 questions of CAGE-AID at baseline
December 2014	Removed safety protocol requirement to contact a patient’s provider in cases of non-imminent suicidal or homicidal ideation
January 2015	• Modified MINI Psychiatric interview question C9 to ask whether patient had made a suicide attempt within past 10 years (instead of lifetime)<br>• Added Secure Messaging option as a

	method of communicating reminders<br>• Changed to administer all static assessments using Microsoft Access instead of Assessment Center<br>• Added exclusion criterion: clinician recommendation of inpatient psychiatric admission within past 3 months<br>• Added option of mailing paper assessments if veteran unable to attend in person
March 2016	• Offered CAPS-5 over the phone if needed for those with a barrier to in person administration
May 2016	• Added exclusion criteria: medical withdrawal from alcohol in past year (if currently drinking), or current drinking with high risk behavior or with past year history alcohol-related seizures or delirium tremens<br>• Narrowing of criteria for an alcohol-related AE to: calculated BAC greater than 0.35.
December 2016	Permission to contact veterans by mail who completed study to inform them we are seeking additional veterans
August 2017	• Increase maximum number of patients randomized from 170 to 204

SPECIFIC AIMS

Posttraumatic stress disorder (PTSD) is a common consequence of trauma that can persist for decades and result in a major reduction in quality of life.[1] Symptoms of PTSD occur in 21 - 31% of soldiers previously deployed to Iraq, 10-31% of Veterans who served in Vietnam, and in similar percentages of victims of assaultive violence and civilian populations exposed to war or acts of violence.[1] It is estimated that the typical person with PTSD experiences symptoms for at least two decades over the lifespan and has a significant lifetime risk of suicide.[2] PTSD often disrupts interpersonal relationships, reduces the ability to work, decreases quality of life, and increases the risk of physical illnesses, substance abuse, and affective disorders.[3]

The VA National Center for PTSD recommends evidence-based pharmacologic and behavioral interventions for PTSD, including medications (selective serotonin-reuptake inhibitors and prazosin), and psychotherapeutic approaches (cognitive therapy, exposure therapy, stress management skills training, and eye movement desensitization reprocessing-EMDR).[4] Cognitive processing therapy (CPT), has been found to be effective in the treatment of PTSD in randomized clinical trials.[5] Despite the availability of these and other interventions, many persons with PTSD continue to experience persistent PTSD symptoms, as well as anger, difficulties with interpersonal relationships, shame, and grief.[6]

Increasing evidence suggests that as many as half of Veterans seen in VA primary care settings report using Complementary and Alternative Medicine (CAM) interventions to address a variety of difficulties[7] and that many do so to limit their use of medications and to ensure that their social and spiritual needs are addressed.[8] A CAM intervention that holds promise for addressing PTSD and depression among Veterans is Loving-Kindness Meditation (LKM). Consistent with Fredrickson's broaden-and-build model of positive emotions, LKM has been shown to broaden individuals' emotional repertoires through increased positive emotions, which mediates the building of a range of personal resources (e.g., improved social support or a sense of environmental mastery), which in turn leads to improved clinical outcomes (e.g., reduced depression).[9] Pilot data from an open trial conducted in our laboratory indicate that LKM is well tolerated by Veterans and is associated with increases in positive emotions, enhanced personal resources, and reductions in PTSD and depressive symptoms.

The proposed study is a randomized, controlled non-inferiority trial that will assess whether LKM results in improvement in PTSD symptoms and depression that are not meaningfully different than Cognitive Processing Therapy—Cognitive only (CPT-C), among Veterans with current PTSD. PTSD symptoms will be measured by the Clinician Administered PTSD Scale - CAPS and depression will be measured by the NIH PROMIS measure.[10] Additional analyses will assess whether mediators of response are consistent with the mechanisms conceptualized for each intervention. One hundred seventy Veterans with PTSD will be randomized to LKM (n = 85) or CPT-C (n = 85). Comprehensive assessments will take place post-treatment and 3- and 6- months later. The following specific aims will be accomplished through the proposed clinical trial:

Aim 1: Evaluate if LKM is non-inferior to CPT-C in producing reductions in PTSD symptoms among Veterans with current PTSD.

Hypothesis 1: Veterans randomized to LKM will report mean reductions in PTSD symptom severity that are not meaningfully worse than CPT-C, as indicated by a lower limit of the 95% confidence interval for difference in 6-month mean CAPS scores that is greater than -10 (corresponding to a standardized effect size of 0.5 between randomization arms). Results will also be assessed at the immediate post-test and 3-month time intervals. *(Addendum: The above description, based on CAPS-IV, was included in the original grant application. However, before the study began, the CAPS-5 became available and was used as the measure of PTSD symptom severity instead. For the CAPS-5, using the same criterion of d =0.5, the non-inferiority margin was defined as 5 points on the CAPS-5. See analysis section for additional details).*

Aim 2: Evaluate the non-inferiority of LKM and CPT-C in producing reductions in depressive symptoms among Veterans with current PTSD.

Hypothesis 2: Veterans randomized to LKM will report mean reductions in depressive symptoms that are not meaningfully worse than CPT-C, as indicated by a lower limit of the 95% confidence interval for difference in 6-month mean PROMIS depression measure that is greater than -4, corresponding to a standardized effect size of approximately 0.5 between randomization arms.[11] Results will also be assessed at the immediate post-test and 3-month time intervals.

Exploratory Aim: Evaluate potential mediators of response to LKM and to CPT to provide preliminary information regarding whether they are consistent with the mechanisms of change conceptualized by each intervention to be associated with improvement in PTSD and depression.

Exploratory Hypothesis 1: Enhanced self-compassion will more strongly mediate PTSD and depressive symptom improvement for those assigned to LKM than those assigned to CPT-C.

Exploratory Hypothesis 2: Reductions in posttraumatic maladaptive beliefs will more strongly mediate PTSD and depressive symptom improvement for those assigned to CPT-C than those assigned to LKM.

BACKGROUND AND SIGNIFICANCE

Posttraumatic stress disorder (PTSD) is a common consequence of trauma that can persist for decades and result in a major reduction in quality of life.[1] PTSD affects approximately 8% of the general population of the US. The clinical hallmarks of PTSD include recurrent, intrusive recollections or reexperiencing of a traumatic event, avoidance of external or internal cues that can trigger reexperiencing, emotional numbing, and hyperarousal.[12] PTSD has been shown to have a greater impact on quality of life than major depression and obsessive-compulsive disorder.[13] Symptoms of PTSD occur in 21 to 31% of soldiers previously deployed to Iraq,[14] 10-31%[15] of Veterans who served in Vietnam, and in similar percentages of victims of assaultive violence and civilian populations exposed to war or acts of violence.[1] It is estimated that the typical person with PTSD experiences active symptoms for at least two decades over the lifespan[1] and has a significant lifetime risk of suicide; PTSD has a stronger association with suicidality than any other anxiety disorder.[2] PTSD often disrupts interpersonal relationships, reduces the ability to work, impairs quality of life, and increases the risk of physical illnesses, substance abuse, and affective disorders.[3] Among individuals seeking treatment for PTSD, the rate of PTSD/depression comorbidity was found to be nearly 50% in the original national comorbidity study sample.[16] Individuals suffering from both PTSD and depression have higher rates of suicidal ideation than those with either disorder alone, though rates of actual suicide attempts are consistent across those with PTSD only and comorbid PTSD/depression, and both groups report significantly higher rates than those with depression only.[17] Comorbid PTSD and depression is also associated with greater risk of various physical conditions than is PTSD alone.[3] Thus, PTSD complicated by co-occurring depression is associated with even greater suffering and compromised functioning than is PTSD alone.

The VA National Center for PTSD recommends evidence-based pharmacologic and psychotherapeutic interventions for PTSD, including medications (selective serotonin-reuptake inhibitors and prazosin), and psychotherapeutic approaches (cognitive therapy, exposure therapy, stress management skills training, and eye movement desensitization reprocessing-EMDR).[4] Selective serotonin reuptake inhibitors (SSRIs) produce improvement in the major symptom clusters of PTSD, although the magnitude of the treatment effect is modest.[18] Prazosin, an alpha-antagonist, reduces trauma-related nightmares and has also been found to have a significant effect on all of the PTSD symptom clusters.[18] Despite these and other pharmacologic agents, many persons with PTSD continue to experience persistent PTSD symptoms, as well as anger, difficulties with interpersonal relationships, shame, and grief.[6] Psychotherapeutic techniques are often combined with pharmacotherapy in the treatment of PTSD. Prolonged exposure (PE) therapy facilitates emotional processing of the traumatic event, helping individuals to develop less phobic responses to internal and external trauma cues.[19] However, PE is difficult for many Veterans to complete, as evidenced by a high dropout rate (38%).[20] PE may also fail to address the entire realm of posttraumatic psychopathology including anger control, interpersonal difficulties or grief.[6] In addition, PE and EMDR are usually administered by an individual therapist rather than as a group treatment, which limits the ability to efficiently deliver these interventions to large numbers of persons. Cognitive Processing Therapy (CPT) can be delivered either individually or in group settings and has some initial support for efficacy in addressing PTSD among Veterans. In addition to concerns about dropout for some Veterans, another potential shortcoming of current treatment for PTSD is the lack of empirical data to guide treatment for persons with multiple psychiatric diagnoses. Psychiatric conditions other than PTSD commonly co-occur with PTSD,[16] yet persons with multiple psychiatric diagnoses have often been excluded from clinical trials. A further limitation is that many studies employed interventions delivered by expert PTSD therapists with extensive experience, raising the question of whether the favorable results from these clinical studies can be generalized.[20]

The VA has invested considerable resources to disseminate both PE and CPT through national roll-outs to increase the likelihood that Veterans will have access to these empirically supported treatments. Although research published in 2004 indicated that neither of these empirically supported treatments were widely utilized by therapists within the VA,[21] more recent findings suggest that the roll-outs have met with initial success and plans are in place to monitor future penetration of PE and CPT within the VA.[22] These efforts will no doubt relieve a great deal of suffering among Veterans with PTSD. However, given the large number of Veterans with PTSD, many with significant comorbidities and not all of whom will benefit from or elect to participate in PE or CPT, additional cost-effective treatments suitable for broad implementation are needed.

Evidence indicates that about 15% of the US adult population utilizes mind-body therapies, with a significantly higher rate found among persons with two or more chronic diseases.[23] Emerging evidence indicates that Veterans also use various complementary and alternative medicine (CAM) interventions at high rates. A large multisite study of VA outpatients found that 27% of Veterans had utilized some form of CAM in the past year.[24] Another study of Veterans found that nearly 50% reported using a CAM intervention, and high daily stress, chronic medical illness and a perceived negative impact of military service on mental or physical health were significant predictors of CAM use.[7] Qualitative research among Veterans also suggests that dissatisfaction with the reliance of conventional care on prescription medications and neglect of social and spiritual aspects of health serve as motivating factors for CAM use.[8] In addition to chronic symptoms of PTSD and depression, the presence of chronic illnesses associated with PTSD along with dissatisfaction with reliance on prescription medications are likely to positively impact recruitment for the current study. Our past experience offering meditation-based interventions as a clinical service to Veterans provides additional evidence that LKM is likely to be an acceptable treatment modality among Veterans.

Loving-Kindness Meditation (LKM) is a CAM approach that facilitates increased positive emotions through meditation exercises designed to develop feelings of warmth, kindness, and compassion for self and others. A growing body of literature indicates that self-compassion is associated with healthy psychological functioning (including life satisfaction and social connectedness), and negatively associated with self-criticism, rumination, thought suppression, anxiety and depression.[25] Self-criticism, rumination and thought suppression are phenomena frequently associated with PTSD, as is depression.[26] Therefore an intervention that increases self-compassion may impact clinical manifestations of PTSD. In a study of PTSD symptoms in university students, greater self-compassion significantly correlated with lower rates of avoidance symptoms, but not re-experiencing or hyperarousal,[27] suggesting that individuals with higher self-compassion may engage in fewer avoidance strategies. 'Compassionate mind training,' which is an intervention designed to teach self-compassion, was offered to six individuals in a small pilot study by Gilbert et al. [28] The authors found significant reductions in depression, self-criticism, anxiety and shame over time, and postulated that teaching techniques that generate feelings of self-soothing and inner warmth may be particularly helpful for persons with a history of trauma, due to pervasive feelings of shame and self-criticism coupled with the relative inability for self-soothing in this population.[28] Many persons with PTSD have a long history of traumatic experiences, and have rarely felt safe or reassured. Pervasive feelings of shame and guilt are common in the setting of PTSD.

Increasing evidence supports LKM as a technique for enhancing positive emotions and health generally. Support for the efficacy of LKM to increase positive emotions was found in a study by Fredrickson and colleagues.[9] This study was conducted with 139 community dwelling individuals employed by a single company. The subjects were randomized to either LKM (n=67) or waitlist control (n=72). Those in the LKM group reported significantly greater positive emotions and were significantly less depressed at the end-point assessment than the no-LKM group, even though both groups reported a similar frequency of negative emotions day-to-day. They also found that positive emotions persisted after meditation sessions ended, and that over time, repeated LKM practice produced a cumulative increase in positive emotions on subsequent days, regardless of whether the individual had practiced meditation on that day.[9] A pilot study of LKM for chronic low back pain by Carson et al compared a group that underwent LKM (n=18) with a group receiving standard care (n = 22) and found that those in the LKM group reported lower pain ratings, less anger, and less psychological distress (note: positive emotions do not appear to have been assessed).[29] Another study of LKM, performed in a group of community participants, indicated that a single brief session of LKM training led to increased self-esteem and sense of social connectedness relative to a control condition.[30] In a report of case studies in which LKM was taught to persons with schizophrenia, LKM appeared to be of potential benefit for persistent negative symptoms.[31]

In LKM practice, a person sits quietly and calls to mind a particular person (e.g., a good friend) and focuses on bringing a sense of positive regard to that individual through a series of standard phrases invoking the desire for safety, happiness, health, and ease or peace for them. Classically, four phrases are used, such as: "may you be safe," "may you be happy," "may you be healthy," and "may your life unfold with ease." Next, the person brings positive regard to other individuals or categories of people, including themselves, neutral persons, and those who have caused difficulty or harm (see Approach section), changing the phrases as needed (i.e., "may you be safe" becomes "may I be safe").[32] This systematic development of kindness toward self and others is intended to change the orientation to one's self, others, one's life experiences, and to result in a broadening of the range of emotional responses and choices available. Loving-kindness practice has its roots in the Buddhist tradition, but as described above an increasing number of studies have successfully applied it as a non-religious practice. The phrase loving-kindness derives from the Pali word metta, which can

be translated as “love” or “unconditional friendliness,” or “loving-kindness,” akin to the Greek word “agape,” which is typically translated as wide open unconditional love. The words loving-kindness are intended to describe an emotional state that is not a sentimental love or a feeling of passion. Rather, it can be described as an unconditional friendliness, benevolence, and openness toward experience – even difficult experience.

It can be postulated that the intentional cultivation of kindness and acceptance promoted in LKM practice will positively influence multiple aspects of recovery from trauma. Although the factors described below might also be developed through mindfulness practice (e.g., in MBSR through breathing meditation), LKM is a distinct form of practice that is often considered more accessible and therefore more helpful to persons who are in significant ongoing distress. Simply said, many persons find it easier, and more helpful, to adopt and stay with a practice of LKM, which relies on the repetition of simple phrases meant to foster kindness and compassion towards self and others, as compared to practicing breathing or body scan meditation. This may be particularly true when an individual is in severe emotional pain. This is in agreement with the recommendations of Buddhist teachers, who have been reported to recommend LKM practice instead of other forms of practice when people are in significant distress.[32] The lives of persons with PTSD are marked by ongoing severe emotional (and often accompanying physical) pain, including the hallmark emotion of fear. In the historical Buddhist record, LKM was originally taught as a way of working with and ‘staying with’ the strong emotion of fear, which, as noted, is the predominant emotional experience in PTSD. Indeed, one of the core features of PTSD is thought to be a phobic response to reminders of a traumatic event and to the memories associated with that event, which in conjunction with information processing problems, lead to pervasive fear and poor functioning.[33] One aspect of LKM practice that can be hypothesized to apply to a key post-trauma sequela is re-establishment of a sense of safety, or basic trust. Trauma can shatter the sense of basic trust in others, oneself, and the world at large. Disruptions in basic trust, as well the tendency to avoid reminders of the trauma in social situations, often result in withdrawal from close relationships.[34] The repetition of LKM phrases can be hypothesized to represent a mechanism to recreate a safe ‘holding environment,’ which in LKM is created by self-directed phrases of positive intention. This may prove particularly helpful when kindness and support are lacking in the environment. Social support has been shown to be an important factor in recovery from PTSD.[35] The incremental restoration of a sense of basic trust, as well as an increased ability to self-modulate pervasive feelings of shame, guilt and inferiority may lead to an enhanced sense of self-efficacy. Over time, enhancement of these factors might be expected to play a role in restoration of a connection to the sense of self that has been lost through traumatic experiences, and may lead to a sense of reconnection with the community as well. The cultivation of positive emotions through LKM might be also particularly helpful for the numbing and constrictive symptoms characteristic of chronic PTSD, which can present as feelings of chronic alienation and deadness. Providing a technique through which positive emotions can repeatedly be enhanced, as in LKM practice, may provide an innovative pathway to address these numbing and constrictive symptoms.

While practicing LKM, a person is asked to notice and feel the positive emotion elicited by each of the phrases of positive intention, or to notice if there is a sense of reluctance, hesitation, or even aversion for the self or another. The practitioner is encouraged to have compassion for these responses and to notice them without judgment. More generally, during LKM, when a person becomes distracted by thoughts that arise during the practice, they are instructed to notice what has distracted them with an attitude of non-judgmental, mindful attention, then return to the LKM phrases and the breath without self-criticism. Walking meditation is also used as a method to teach LKM. Informal LKM practices are also encouraged - participants are asked to practice LKM toward themselves or others during everyday activities such as walking, eating, washing the dishes, or standing in line at the grocery store.

LKM and the Broaden-and-Build Theory of Positive Emotions

The broaden-and-build theory of positive emotions provides a framework for understanding the potential influence of LKM practice on affect, behaviors, and quality of life.[9,36] The broaden-and-build theory proposes that positive emotions momentarily broaden a person’s attention and thinking, and with this broadened outlook they are able to build personal resources. A key postulate in the broaden-and-build theory is that positive emotions broaden a person’s thought-action repertoire.[36] As described by Fredrickson: “Joy sparks the urge to play, interest sparks the urge to explore, contentment sparks the urge to savour and integrate, and love sparks a recurring cycle of each of these urges within safe, close relationships.”[36] The broadened mindset associated with positive emotions is contrasted to a narrow focus often associated with negative emotions, and characterized by specific, repetitious thought-action tendencies (e.g., fight or flight). A second key postulate of the broaden-and-build theory is that the broadened mindset associated with positive emotions leads to the building of enduring personal resources. These personal resources are thought to arise

through new thought-action sequences such as play, curiosity, and openness to new situations and ideas, as well as new social interactions. Personal resources built may include cognitive resources (e.g., the ability to mindfully attend to the present moment), psychological (e.g., a sense of purpose in life, self-acceptance or self-compassion, or a sense of environmental mastery) social (e.g., improved ability to give and receive social support) or physical (e.g., reduced susceptibility to stress-associated illnesses).[9] According to the broaden-and-build theory, people who have developed these personal resources are more likely to successfully navigate life's challenges and live happier, healthier lives.

There is growing evidence in support of the broaden-and-build theory. The 'broaden' hypothesis predicts that positive emotions broaden a person's attention and thinking. Support for this hypothesis comes from studies of experimentally-induced positive emotional states (through LKM in some cases as well as through other means), which show that positive emotions broaden the scope of a person's visual attention, increases one's openness to new experiences, improves ability to accept critical feedback, improves ability to recognize persons of another race,[37] broadens one's repertoire of desired actions, and enhances a sense of connection with others.[9] In a randomized controlled trial of LKM vs. a waitlist control (described in detail above), the findings supported both the 'broaden' aspect (enhanced positive emotions) as well as the 'build' part of the theory (enhanced personal resources).[9] When life satisfaction was assessed as an outcome, positive emotions predicted life satisfaction only to the extent that they built personal resources.[9] In addition, when depression was assessed as an outcome, LKM was found to have direct effects on depressive symptoms as well as additional effects mediated by built personal resources.[9] The built cognitive, psychological and social resources appear to have enhanced the ability of persons randomized to LKM to ward off symptoms of depression. Similarly, in the proposed trial, we hypothesize that personal resources will be built as a result of LKM practice, which will positively influence both depressive and PTSD symptoms.

Mediators

Identification of active components of treatments is a priority for treatment research.[38] The proposed study will evaluate potential mediators of change thought to be specific to each intervention.

LKM: We will evaluate whether enhanced self-compassion mediates change in PTSD and depression for LKM. Self-compassion can be considered a healthy form of self-acceptance. Neff defines self-compassion as comprised of three facets: (1) treating oneself kindly and without harsh judgment in the setting of pain and failure, (2) perceiving that mistakes and hardships are part of the common human experience rather than isolating, and (3) maintaining mindful, non-judgmental awareness of thoughts and feelings rather than or overidentifying with them.[39] Self-compassion is distinct from self-esteem, which is typically defined as an evaluative process in which one's abilities and characteristics are compared to standards.[39] Self-compassion is also distinct from self-centeredness, because self-compassion acknowledges the ubiquity of pain and hardship among humans. LKM is considered a method to enhance self-compassion. As suggested by our pilot work with LKM, we hypothesize that for those in the LKM arm of the study self-compassion will mediate improved outcomes for persons with PTSD such that changes in self-compassion will both temporally precede changes in PTSD and when they are statistically controlled, the changes in PTSD will be attenuated. There is initial support for this hypothesis from the literature.[40,41] In an analysis of people with a history of major depression randomized to a mindfulness intervention or maintenance antidepressants, enhanced self-compassion mediated the relationship between participation in mindfulness training and reduced depressive symptoms at follow-up.[41] The possibility of a key role for self-compassion is consistent with emerging theory and evidence that self-compassion is adaptive in the setting of painful or difficult thoughts and feelings. [28,40] It is also possible that changes self-compassion will mediate changes in PTSD for those in the CPT-C arm of the study, but we anticipate that these effects will be less robust than for those in the LKM arm of the study.

CPT-C: We will evaluate whether reductions in posttraumatic maladaptive cognitions mediates improvement in PTSD and in depression for CPT-C. CPT is hypothesized to facilitate improved emotional processing through repeated practice restructuring dysfunctional beliefs and appraisals of situations that are related to the individual's trauma history, which lead to painful affect and reactive behavioral choices.[42,43] Reduction of posttraumatic maladaptive beliefs is postulated to be a key ingredient in CPT that will mediate improvement. Indeed, recent work evaluating whether cognitive change predicts symptom reduction in the context of cognitive therapy found support for this idea. Analysis of weekly measures collected during a course of cognitive therapy found that changes in posttraumatic cognitions preceded and predicted changes in PTSD symptom severity and that changes in PTSD symptom severity did not precede or predict changes in posttraumatic cognitions.[44] Although this work was done in the context of an alternate cognitive therapy intervention, Cognitive Therapy for PTSD, and not CPT, the finding suggests that CPT is also likely to result in

such changes in beliefs and appraisals that in turn mediate PTSD symptom reduction. The proposed project would afford the opportunity to evaluate whether this is the case for Veterans who participate in group-based CPT-C. [45] We will also be able to assess whether reductions in maladaptive cognitions also mediates reductions in PTSD for those who receive training in LKM.

The Approach: What LKM Is and Isn't

Having provided an overview of what LKM is, we also believe it is important to delineate it from other approaches with which it may be confused. First, although both LKM and mindfulness meditation practices involve sitting meditation, typically with closed eyes and an initial focus on the breath, they differ in several respects. Mindfulness meditation cultivates the ability to pay attention, without judgment in the present moment, whereas LKM specifically develops the ability to experience kindness, warmth, and openheartedness toward self and others. The intentionality of LKM is toward developing the ability to experience positive emotions, rather than developing non-judgmental awareness. Second, LKM differs from intercessory prayer. Although in LKM practice the phrases repeated express the intention of wellbeing for self and others (see below), it is made clear that there is no expectation that the LKM phrases will actually benefit others. Instead, it is made explicit that the goal of LKM practice is to benefit oneself, such that in developing the capacity to experience kindness toward self and others, the person who holds this intention actually benefits. Unfortunately the intercessory prayer literature has not evaluated whether praying for others has a salutary effect on the people doing the praying, though research on spiritual involvement, including prayer activity, has found that Veterans with greater spiritual involvement have better physical and mental health than those with less spiritual involvement.[46] Third, although LKM involves the repetition of phrases, it is distinct from mantram repetition practices, which involve bringing attention to a repeated phrase or word that facilitates disengagement from difficult thoughts and feelings, and encourages relaxation. In LKM, phrases of positive intention are utilized as a means to bring forth compassion for self and others, and to foster a broadened emotional repertoire. Through the process of practicing LKM with different people in mind, LKM is thought to reshape relationships with others, as well as to foster self-compassion. Of note, an additional effect of LKM is that in the process of choosing phrases of positive intention (which carry personal meaning for a participant), clarification of values and one's intentions occurs. LKM is also distinct from practices explicitly intended to promote forgiveness, although forgiveness may occur through LKM practice. For the purposes of scientific study, we have chosen to not include explicit forgiveness practice as part of our study. In LKM, the practice is intended to develop the ability to look at others and see oneself, and in so doing, develop self-compassion and compassion for others. An expanded understanding of the commonality of human experience, such that all humans suffer and all desire happiness, which is fostered by LKM, is likely to lead to forgiveness. In addition, LKM is also distinct from the practice of verbalizing positive affirmations. While there may be some overlap with regard to the shared emphasis on positivity, affirmations tend to be stated as though they are definite personal qualities and are intended to strengthen self-confidence (e.g., "I am a good, kind person"), whereas LKM is usually stated as an invitation or request that is intended to tap into the intention for kindness and compassion for oneself and for others (e.g., "may I be safe from harm;" "may you be happy and healthy"). Importantly, another way that LKM is distinct from positive affirmations is that LKM is a meditation practice intended to broaden attention and allow positive emotions to remain in awareness for sustained periods of time. Finally, LKM is not intended to be used to remove painful thoughts or feelings. Rather than attempting to rid oneself of these experiences, it is anticipated that individuals with PTSD can learn through LKM that pain can co-exist with positive feelings because, after all, it is part of being human to experience both. This may help address the phenomenon that we have frequently observed clinically wherein individuals with PTSD are afraid to experience positive emotions because it is painful when they end. By re-learning (or learning for the first time) that it is safe to feel good and that it is normal to have good feelings (and painful ones) that wax and wane, the often profound emotional numbing typically associated with PTSD may be mitigated.[47]

Overview of Proposed Interventions

Intervention #1: Loving-Kindness Meditation (LKM)

The LKM course follows the instructions for LKM as described by Salzberg.[32] During the12 meetings the ten to twelve group participants will receive instruction from two expert meditation teachers (who will co-teach the sessions) in the practice of LKM, including both in-session practice of LKM and group discussions. The intervention is designed to allow patients to adopt skills and techniques they can continue to practice without the need for an ongoing relationship with a therapist. LKM is taught in a non-religious format. A class-by-class outline is provided in the participant workbook (Appendix 3).

In order to help develop concentration, a necessary component of LKM practice, the first two sessions focus on cultivating mindful attention of one's breath. The primary LKM practice begins in week 3, and involves

sitting in a comfortable, relaxed position with closed eyes or a neutral visual focus and then bringing to mind various categories of beings – self and others. The meditator is then asked to gently repeat phrases to the person who he or she has brought to mind; these phrases are intended to have meaning in terms of what is wished for that person. In order to facilitate stepped learning, the class begins their LKM practice focusing on a positive benefactor and over the remaining nine weeks other individuals and categories of individuals are introduced, including the self and people who have caused the participant difficulty or harm.

Intervention #2: Cognitive Processing Therapy (CPT). The CPT intervention is based on Patricia Resick and colleagues' manual for treating PTSD among military veterans.[48] This version of CPT highlights both combat traumas and interpersonal violence and is therefore more appropriate for our population than the original version, which focuses on adult rape survivors. (See CPT manual: Appendix 4) CPT evolved directly out of Social-cognitive Information Processing Theory (SCIPT), which posits that deficits in emotional and cognitive processing of traumatic events lead to the development of PTSD.[48] The fundamental assumption of the theory is that post-trauma psychopathology occurs when there is a failure to integrate perceptions of the traumatic event with preexisting beliefs.[49] SCIPT posits recovery from a traumatic event occurs when beliefs affected by the traumatic event are integrated with prior beliefs.[49] Recovery happens when beliefs affected by the event are accurate and realistic and negative affect about the trauma is processed. Avoidance symptoms, according to this theory, maintain the other symptoms, in that they both prevent the memory of the traumatic event from being integrated with prior beliefs, and prevent the examination of inaccurate beliefs affected by the traumatic event.

CPT combines cognitive restructuring with emotional processing of trauma related content. The therapy sessions initially focus on rigid or inaccurate beliefs about the traumatic event itself, which often reflect self-blame or hindsight bias. Later sessions address over-generalized beliefs about the self and others that result from a traumatic event. CPT focuses on over-generalized trauma-related beliefs relevant to five key areas: safety, trust, power, esteem, and intimacy. Over the course of treatment, clients learn to identify and modify these beliefs in order to develop more balanced, flexible, and ultimately, more adaptive beliefs.

Cognitive processing therapy (CPT), has been found to be effective in the treatment of PTSD in randomized clinical trials conducted by different research teams and with different trauma exposed populations, including survivors of sexual and physical assault[50] and combat Veterans.[51] In these trials, CPT was effective in reducing symptoms of PTSD and depression for up to 5 to ten years.[50] CPT has also been found to be effective in reducing negative emotions such as grief, shame, guilt and anger.[50,51] Based on these randomized clinical trials, CPT is being widely disseminated, including to the US Veterans' Administration (VA) mental health system.

A version of CPT that does not include writing a trauma narrative has also been evaluated, CPT-C, and has been found to be associated with more rapid improvement than standard CPT-C as well as comparable outcomes to standard CPT.[43] The elimination of the trauma narrative makes CPT-C more conducive to a group-based delivery platform as it reduces the risk that group participants will be traumatized by one another's stories. CPT-C has recently been shown to be effective in a group format for Veterans, active duty military personnel, and reservists with PTSD.[5] In a randomized controlled trial (N=107), group CPT-C was compared to present-centered therapy (PCT) and the former found to be more effective than the latter in reducing PTSD and depressive symptoms in active duty military personal with PTSD. Between group comparisons showed that the CPT-C group had significantly greater improvement in PTSD symptoms than PCT with a medium effect size ($d=0.42$; $p = 0.03$).[5]

Importance of Study Findings to VHA: The demand for mental health services provided by VHA has grown dramatically in recent years - more than a half millions Operations Iraqi Freedom and Enduring Freedom (OIF/OEF) Veterans have sought VHA care while the number of Vietnam Veterans receiving VHA mental health services has doubled over the past decade.[52] PTSD is highly prevalent among Veterans, with prevalence rates [13]of approximately 20 - 30% of combat Veterans.[14] [15] A study of approximately 104,000 OIF/OEF Veterans found that 25% received a mental health diagnosis when they accessed VA health care, and of these, 13% were diagnosed with PTSD.[53] To respond to the growing demand for mental health services, consensus recommendations have identified the need for further research on nontraditional delivery systems and group-based interventions to significantly expand the availability of cost-effective therapies.[54] Given the large number of persons with PTSD, not all of whom will opt for or benefit sufficiently from PE or CPT, additional cost-effective treatments suitable for broad implementation are needed that address persistent symptoms and reduced QOL. Thus, if LKM is shown to be non-inferior to CPT, it would represent a novel, and likely cost effective, approach to mental health care delivery.

A key tenet of patient-centered care is that health care practitioners listen to and honor patient and family perspectives and choices. Patient and family knowledge, values, beliefs, and cultural backgrounds are incorporated into the planning and delivery of care. This study would provide information to help patients decide whether a contemplative practice, such as LKM meditation, presents a viable alternative to a trauma-focused behavioral intervention such as CPT-C.

APPROACH

Design Overview and Rationale

The proposed study is a randomized controlled non-inferiority trial comparing Loving-Kindness Meditation (LKM) with Cognitive Processing Therapy – Cognitive only (CPT-C) for treatment of PTSD and depression among Veterans recruited from the VA Puget Sound Health Care System. Veterans (N = 170) with PTSD will be randomized to receive 12 weeks of either LKM (n = 85) or CPT-C (n = 85). The two interventions are structurally similar - Each intervention meets for 90 minutes per week for twelve weeks; the overall amount of treatment time is the same across the two interventions. Both interventions will be delivered to groups of Veterans by experienced leaders, and fidelity coding from audiotapes will evaluate protocol adherence. Participants will complete brief take home paper assessments weekly for the 12-week duration of the active treatment phase to assess key outcomes, potential mediators of change, and completion of homework assignments. All participants in each study condition will complete an in-person baseline assessment, which will be repeated immediately after the treatment phase as well as 3- and 6- months post-intervention. In-person assessments will be performed by a blinded assessor.

Use of an Active Control Arm: The active control will account for the non-specific elements of LKM (e.g., group support, positive expectancy). The proposed control arm is CPT-C, which will provide the same amount of clinician-exposure time (it is also a group-based, 12-week duration, 90-minute per session program). Similar to LKM, CPT-C is taught by experienced instructors who believe in the benefit of the program; allegiance of the researcher/therapist has been shown to be strong predictor of treatment outcomes.[55] CPT-C therefore contains key elements of an active control – it is structurally similar to LKM (thus controlling for non-specific elements of the intervention) and given that it is an accepted PTSD treatment, CPT-C will foster positive expectation for intervention success by both therapists and patients. Each of these elements has been described as necessary for an appropriate active control.[56]

Settings, Recruitment, and Participants

Settings. The study's clinical activities will take place at VAPSHCS; Seattle Division. In FY10 over 17,700 Veterans with chart diagnoses of PTSD received care at VAPSHCS and over 10,000 of them had a mental health stop code, indicating that the majority were likely invested in addressing their PTSD symptoms. VA Puget Sound has the nation's largest clinical program for PTSD.

Recruitment. The primary mechanism of recruitment will be letters sent to Veterans with a diagnosis of PTSD (as identified using a VA database called the Corporate Data Warehouse; CDW). To illustrate the effectiveness of this method, we are currently conducting a trial of MBSR for Gulf War Syndrome (see preliminary studies) for which we are already successfully recruiting at the same rate as for the proposed RCT. Thus, the goal of recruiting 8 Veterans per month with PTSD is feasible given the large pool of Veterans with PTSD.

Participants. Based on our experiences recruiting individuals with PTSD for LKM and MBSR research, we anticipate that we will need to conduct phone screens with approximately 350 individuals and in-person screening. We anticipate that we will enroll at least 250 male and female Veterans over age 18 with a current DSM-IV diagnosis of PTSD enrolled at VAPSHCS who will provide informed consent and undergo in-person screening. Of these, we anticipate that 170 individuals with PTSD who meet study criteria will be randomized. Gender and ethnic/racial distribution of the participants is expected to mirror that of our prior PTSD MBSR studies in which similar recruitment strategies were used; that is 40% female, 79% Caucasian, 8% African American, 6.5% Hispanic, and 6.5% other.

Inclusion and Exclusion Criteria. Inclusion criteria: current DSM-5 diagnosis of PTSD, age ≥ 18 years, and English fluency. Participants must be willing to not participate in Mindfulness-Based Stress Reduction (MBSR) or Prolonged Exposure (PE) therapy during the study period. Exclusion criteria: current substance use dependence disorder other than alcohol; alcohol involvement that poses a safety concern (i.e., currently drinking and has a past year history of alcohol-related seizures or delirium tremens), suicide attempt or suicidal ideation with intent or plan, self-harm in the past month, a psychotic disorder, uncontrolled bipolar disorder, chart diagnoses of borderline personality disorder, antisocial personality disorder, or dementia in-patient

admission for psychiatric reasons within the past month, prior participation in LKM, or CPT. Medication, supportive individual or group counseling, case management, and self-help programs will be allowed concurrently and assessed as potential covariates.

Sequence of Study Visits

Telephone Screen. Veterans who contact the Project Manager will be provided an overview of the study. Veterans who remain interested will undergo a 15-20-minute telephone screen to ascertain basic inclusion/exclusion criteria.

In-person Screen and Baseline Assessment. Following informed consent, participants will undergo a 2-hour assessment. The Study Assessor will conduct standardized interviews for the presence of exclusionary disorders (relevant subsections of the MINI International Neuropsychiatric Interview) and for diagnosing PTSD (Clinician Administered PTSD Survey; CAPS).[57] Self-report measures will be administered on a VA-approved computer. Those who are eligible to continue the study will receive their randomization assignment within approximately one week and will begin either LKM or CPT-C within one month.

Weekly Paper Assessments. Participants will complete brief measures of PTSD and depressive symptomatology, the hypothesized mediators of change (self-compassion and maladaptive posttraumatic cognitions), and homework activity for 12 weeks during the active treatment phase.

Follow-up Assessments. Participants will complete a post-treatment assessment within a week of completing LKM or CPT-C, and will repeat the assessment battery 3- and 6-months thereafter.

Randomization

We will utilize a blocked randomization approach stratified by symptom severity on PTSD because our pilot studies indicate that those who score in the severe range (60 or higher)[57] on the PCL decrease their PTSD symptoms more than those with less severe PTSD but are still more symptomatic at immediate post-test. Baseline severity of PTSD will be dichotomized based on CAPS-5 scores $\geq$ 37 Because our pilot data did not suggest that severity of depression moderated outcome, we will not include that in the randomization stratification. Similarly, we chose not to stratify on gender because our pilot data indicate that this factor is not predictive of PTSD symptoms at follow-up. A series of randomization tables will be constructed and will be maintained by the research coordinator. Subjects will be randomized in small blocks of varying size using concealed allocation, after the baseline assessment and within two weeks before the next study cohort is set to begin treatment.

Study Interventions:

#1 LKM Course Overview

Each class meeting will begin with either a brief mindfulness meditation (weeks 1 and 2) or an LKM meditation followed by an opportunity for participants to discuss their experiences integrating LKM class material into their daily lives. New material will then be presented, typically introducing a new category of beings (i.e., benefactor, oneself, strangers, etc.) and particular challenges associated with working with that category of beings will be discussed. The class will then practice LKM incorporating the new category and then discuss the experience. At the end of each class the homework for the next week will be presented and class will end with another brief LKM practice. Participants will be provided with an LKM meditation CD and a workbook to accompany the class. (See appendix 3, LKM manual, for additional details). An overview of the class schedule is provided below:

Classes 1 and 2 provide an introduction to in mindfulness meditation.

Class 3 introduces LKM, including the LKM phrases and a description of the meaning of each phrase. Begin LKM practice toward a benefactor, which is defined as a person who has been kind or helpful and for whom there is gratitude, and respect. Participants will be asked to recall the ways this benefactor has helped them, and the goodness within this person.

Class 4 introduces the theme of commitment to our own happiness as a basis for intimacy and connection with self and others. Exercise: “Remembering the good within you” (Instructions: “For 10-15 minutes call to mind something you have said or done that was a kind or good action”). Acknowledgement and discussion of the finding that LKM toward our self is often difficult. If participant is unable to practice LKM toward self, suggestion of the concept that underlying all action is a desire to be happy – this may provide a method of noticing kindness toward oneself that already exists; this may act as a starting point for feeling positive emotion for oneself. LKM toward benefactor and self with the 4 phrases.

Class 5 begins with a discussion of homework - LKM in the previous week toward a benefactor and self. Introduction of walking meditation and the concept of a beloved friend (may be a person or an animal).

Class 6 begins with a discussion of how it is often more difficult to practice LKM toward self as compared to a benefactor or beloved friend. Examination of how our life experiences up to this point may affect our experience practicing LKM.

Class 7 introduces the concept of a neutral person – someone we do not strongly like or dislike. Perhaps this is a person we do not know well. This may bring up the awareness that this may be difficult because of the judgments we hold for people around us as well as pervasive fears and mistrust.

Class 8 begins with a discussion of homework - LKM to a neutral person. Have your feelings for that person changed? Introduction of a difficult person: a person with whom we have experienced conflict and toward whom we feel anger or lack of forgiveness. This could be someone who has been unsupportive or who responded to us in ways that were not helpful when we needed them. At this stage, participants will be instructed at this stage to not choose the person who has caused them the most pain or suffering in their lives – while acknowledging that this is a suggested goal of LKM practice. Discussion of how practicing LKM does not mean a sentimental love toward that person, as well as a discussion of anger – how it can serve the purpose of setting boundaries and can challenge injustice, but can also cause suffering for the person who holds anger as well as for the person who receives it.

Class 9 introduces the difficulties involved in LKM to a difficult person. Discussion: When you are feeling anger, who is the person suffering from anger? Include discussion that LKM toward difficult person is actually compassionate toward oneself, because it promotes letting go of this anger. Introduction of concept of difficult aspects of oneself, including painful feelings, thoughts, memories, and symptoms. What aspects of ourselves do we reject and feel anger or hatred toward?

Class 10 provides opportunity for further discussion of LKM toward difficult person. Introduction of LKM toward groups. Discussion of how we often accumulate biases or prejudices toward groups or categories, and these biases are often not in our conscious awareness. Practice LKM toward complementary sets of groups, with discussion of whether participants feel an affinity or sense of unease practicing LKM toward one part of a group. Examples of groups include: males/females, young/elderly, Veteran/civilian, all those known to me/all those unknown to me, those near/those far.

Class 11 begins with discussion of LKM toward groups, with an emphasis on whether unconscious affinity or bias was detected in the ability to practice LKM.

Class 12 begins with discussion of walking meditation. Introduction of LKM for all beings. Discussion of different categories of beings as appropriate for each person – alive or dead, human or animal. Practice LKM toward self, friend, benefactor, neutral person, difficult person, groups, toward all beings.

Homework for those randomized to LKM homework will consist of 30 minutes of sitting or walking mindfulness (first two weeks) or LKM meditation facilitated by CD recordings with new categories of beings added as described above. Participants will also be instructed to identify opportunities for informal LKM practice each week.

#2 CPT-C Course Overview.

CPT-C will be led by clinicians who will be contracted by the grant to deliver this service. The group co-leaders will all have completed the VA's CPT-C training and certification program or the equivalent in the community. In addition, these clinicians will go through the necessary background checks and credentialing procedures prior to having contact with any Veterans.

Those assigned to CPT-C will attend weekly 90 minute group therapy sessions for 12 weeks. Treatment will be provided in groups of 10 to 12 male and female Veterans with PTSD.

Session 1 provides an introduction to CPT-C and education about both PTSD as a response to trauma and how trauma exposure can lead to distorted thinking and beliefs. Patients are oriented to the Impact Statement homework.

Session 2 draws from the Impact Statement homework and reviews how patients are currently framing the meaning of their index trauma with regard to its impact on their lives in the following areas: safety, trust, power/control, esteem, and intimacy. The concept of trauma-related cognitive "stuck points" is introduced.

Session 3 introduces a basic cognitive therapy overview of the relationship between thoughts and feelings prompted by triggering events through the ABC sheet (A = activating event; B = beliefs; C = consequences).

Session 4 elaborates on the idea of "stuck points" (e.g., "I am damaged goods;" "I'm a failure because I couldn't save my best buddy," etc.) that may have arisen as a result of the trauma exposure and each group member is assisted in identifying their own specific "stuck points."

Session 5 provides a standard list of challenging questions that patients use to help themselves identify stuck points.

Session 6 provides an overview of common patterns of problematic thinking (e.g., all/none thinking, confusing feelings with facts, etc.). The Challenging Beliefs Worksheet is introduced in this session.

Session 7 provides further instruction on the use of the Challenging Beliefs Worksheet.

Session 8 reviews the safety module and patients are instructed to focus on at least one safety-related issue when completing worksheets for homework.

Session 9 reviews the trust module and patients are instructed to focus on at least one trust-related issue when completing worksheets for homework.

Session 10 reviews issues related to power/control and patients are instructed to focus on at least one power/control-related issue when completing worksheets for homework.

Session 11 reviews self- and other-esteem issues and patients are instructed to focus on at least one esteem-related issue when completing worksheets for homework and to write their final impact statement.

Session 12 reviews intimacy issues and the patients' current sense of the event's meaning.

Homework for those randomized to CPT-C will consist of 30 minutes of CPT-C-related homework 6 days a week including writing an impact statement at the beginning and the end of treatment, completing CPT-C worksheets, and completing exercises regarding safety, trust, power/control, esteem, and intimacy.

Procedures for Maximizing Research Integrity and Patient Safety

LKM Fidelity. The LKM teachers are highly experienced meditation teachers with experience teaching meditation to Veterans with PTSD at VAPSHCS. These teachers currently teach LKM groups and Mindfulness-Based Stress Reduction (MBSR) groups at VAPSHCS, which are offered as hospital-wide clinical programs. These teachers have extensive personal experience involving LKM. LKM is also included as a small component of the MBSR program, although the primary focus of MBSR is on mindfulness meditation. Each of these teachers is employed as a WOC-status contract employee, and meets stringent foundational teacher training requirements for MBSR, which we also apply to LKM teaching requirements: longstanding practice of mindfulness and loving-kindness meditation, experience with a body-centered awareness practice, attendance at silent mindfulness meditation retreats of at least 7 days duration, completion of the University of MA Center for Mindfulness Residential Training/Retreat as well as a teacher practicum in MBSR. We believe that these teacher training requirements and prior experience teaching MBSR will be directly applicable to high-quality teaching of LKM.

To assure quality control, Dr. David Kearney will review at least 2 early session tapes from each LKM cohort to provide supervisory feedback. Teaching responsibilities will be shared by Carolyn McManus, PT, MA and Jonas Batt, MA, MHC, both of whom currently teach LKM at VAPSHCS. They will co-teach the LKM courses to provide different perspectives and gender balance across the facilitators. If either of these teachers becomes unavailable, there are 3 additional experienced mindfulness and loving-kindness meditation teachers who are currently teaching other meditation classes at VAPSHCS who will be available to teach LKM. A subset of 20% of LKM classes will be coded for protocol adherence by two independent raters (see Appendix 5). Adherence and competence will be measured using treatment fidelity ratings based on the LKM curriculum. Our pool of undergraduate raters will be trained by Dr. Kearney, who will provide ongoing supervision regarding fidelity ratings.

CPT-C Fidelity. CPT-C group leaders selected to provide CPT-C will have completed either the VA's national rollout dissemination training and certification process or the community equivalent, and thus will have had at least 6 months of supervised experience delivering CPT-C. They will also be employed as WOC contractors. The CPT-C supervisor will be Dr. Carie Rodgers is a national trainer in CPT for the VA National Center for PTSD who was trained by CPT originator, Dr. Patricia Resick and has extensive experience both delivering group-based CPT-C herself within VHA and supervising others.. She will provide weekly supervision based on both review of session tapes and oral reports from the group co-leaders to assure real-time quality control of CPT-C delivery.

A random subset of 20% of each group's 12 sessions will be coded for protocol adherence and competence by two independent raters from our pool of undergraduate raters (see Appendix 6 for coding forms). Adherence and competence will be measured using established published measures of CPT-C

treatment fidelity. Raters will be trained by Dr. Galovski (CPT-C) with ongoing supervision provided by Dr. Rodgers.

CAPS Interview Fidelity. The Study Assessor will a be master's level clinician who will undergo training on the CAPS interview by Dr. Simpson. Dr. Simpson will sit in on at least 5% of the CAPS interviews to provide ongoing supervision.

Maintaining the Assessment Blind. The Study Assessor will be kept blind to participant condition: participants will be reminded not to disclose treatment assignment, and other study personnel will avoid communications that could provide such information.

Procedures for Maximizing Retention. Participants will update contact information at each visit and will be paid $20 for baseline, post-test, $30 for the 3- and 6-month assessments, and $5 per week for the 12 online assessments. Participants who complete all assessments will be eligible for an additional $20 bonus.The maximum remuneration is $180 per enrollee. Frequent contact maximizes participant retention.[58]

Project Management. We will utilize web-based software to ensure coordination among study team members and reliable participant tracking.

Electronic Data Capture. We will utilize the online version of Assessment Center that conforms to VHA privacy and security requirements to create user friendly electronic versions of our measures that participants will complete either using VA desktop computers during the in-person major assessment appointments. This method of collecting data will ensure that items that are skipped are intentionally skipped, thus cutting down on missing data. It will also reduce the need for manual data entry, thereby decreasing the risk of introducing human error into the dataset and increasing the efficiency of moving from data collection to data cleaning and analysis.

Maintaining Participant Safety. Participants will be provided a phone number to call if they require psychiatric assistance, as well as the local county crisis clinic phone number. See also Appendix 7 for a full description of safety procedures.

Study Instruments and Materials (Table 3; see Appendix 8 for study measures)

Study Construct/Variables	Study Phase	Measurement Scale	Purpose
**Inclusion/Exclusion Criteria**			
Demographic information	B		sample description, blocking (gender); moderators
MINI International Neuropsychiatric Interview V-5 (DSM-IV)[59]	B	dichotomous	sample description, exclusion
Medical history interview (seizures, DT's)	B	dichotomous	exclusion
Question assessing ideation of harm to self or others	B		exclusion
Drug Card Sort	B		exclusion
CAGE-AID	B		exclusion
**Tracking**			
Contact form	B,P,3,6		retention
**Primary Outcomes**			
Life Events Checklist[60]	B,P,3,6	dichotomous	inclusion (establish Criterion A)
Clinician-Administered PTSD Scale (CAPS)[57]	B, P,3,6	continuous	inclusion, blocking, **primary outcome**
NIH PROMIS depression measure[10]	B,P,3,6	continuous	**primary outcome**
**Mediation Analyses**			
Self-Compassion Scale-Short Form[61]	B, W,P,3,6	continuous	mediation analyses
Posttraumatic cognitions inventory[62]	B,W,P,3,6	continuous	mediation analyses
PTSD Checklist; Civilian Version (PCL-C)[63]	B,W,P,3,6	continuous	mediation analyses, secondary outcome measure
NIH PROMIS depression measure (static version-SF8a)[10]	W	continuous	mediation analyses, secondary outcome measure
**Other**			
Chronic Multisymptom Illness (CMI)	B		

Survey			
Spiritual Transcendence Index	B		
Rome III IBS Criteria	B		
TBI screening questions	B		
Cognitive Failures Questionnaire	B, P, 3, 6		
AUDIT-C	B		
DAST-10	B, P, 3, 6		
NIH PROMIS alcohol item bank: Use, Negative Consequences	B, P, 3, 6		
Alcohol Consumption (for BAC) short form	B, P, 3, 6		
Experiences Questionnaire	B, P, 3, 6		
Veterans Short Form Health Survey	B, P, 3, 6		
NIH PROMIS CAT measures: pain interference, fatigue, sleep disturbances	B, P, 3, 6		
NIH PROMIS Global Health, short form, static	B, P, 3, 6		
NIH Toolbox Emotion Battery	B, P, 3, 6		
Average daily pain intensity	B, P, 3, 6		
Curiosity subscale of Toronto Mindfulness Scale- short form	B, P, 3, 6		
PROMIS GI Symptom Scales	B, P, 3, 6		
Five Facet Mindfulness Questionnaire- short form	B, P, 3, 6		
Medication usage	B, P, 3, 6		
Qualitative Interview	3		
**VA mental health care**			
Corporate data warehouse mental health psychotherapy and medication management stopcodes	9-month post-baseline interval	dichotomous & continuous	check on VA mental health services received by both conditions
CPRS review for engagement in mental health treatment	9-months post-baseline	dichotomous	check on whether either PE or CPT was obtained during study

Baseline (B), Post-test (P), Weekly, 3- and 6-month Assessments

Overview of Outcome Measures Chosen, and Further Description of Measures

We have chosen the following measures in order to assess outcomes for the aims of this study, including PTSD symptomatology, depression, and potential mediators. The weekly monitoring phase will record the amount of homework practice completed (for both LKM and CPT-C), in order to provide the ability to assess whether homework practice is predictive of improved outcomes.

<u>PTSD Symptoms.</u> Participants' PTSD symptomatology will be assessed using the Clinician-Administered PTSD Scale (CAPS) and the PTSD checklist (PCL).

The **CAPS** is the primary outcome measure. The CAPS requires a clinician to rate 17 diagnostic symptoms of PTSD, as defined by DSM-IV criteria. (Note: because a new version of the DSM is due out in May 2013, we will update our inclusion/exclusion criteria and the main outcome assessment materials once DSM-V is made available). The CAPS gives both a continuous measure of PTSD severity and a dichotomous diagnosis of PTSD. The psychometric properties of the CAPS have been evaluated in several studies and have been found to be excellent.[57] The CAPS takes approximately 45 minutes to complete and will be administered at baseline, week 8, and at each major assessment (post-LKM/CPT-C, 3- and 6-months).

Addendum: The CAPS-5 version of the CAPS measure became available before initiation of the study and was used instead of the CAPS-IV. The CAPS-5 was used to assess PTSD diagnostic status and PTSD severity. The CAPS-5 is a 30-item structured interview (range 0-80).[64] It has high internal consistency (α=0.88), high interrater reliability (ICC=0.91), and good test-retest reliability (ICC=0.78).[64] Interviews were performed by an experienced clinician (M.S.) blinded to participant condition.

The PCL-C is a 17-item self-report measure that correlates highly with scores derived from the Clinician Administered PTSD Scale (CAPS).[63] The PCL has good internal consistency, item-total correlations, concurrent and convergent validity, and test-retest reliability. Each item is rated by the patient on a scale of 1 to 5, and the total score of the PCL is calculated as the sum of the all the items. Higher scores reflect more

severe PTSD.[59] The PCL will be administered during the weekly monitoring phase in order to allow assessment of clinical outcomes as part of mediation analyses, and will be included at weekly time points (post-LKM/CPT-C, 3- and 6-months) as a secondary outcome measure.

Addendum: The 20-item PCL-C version that corresponds with DSM-5 PTSD criteria became available before initiation of the study and was used instead of the 17-item version

Traumatic events. The number and type of traumatic events sustained over the course of each the Veteran's life will be assessed using the **Life Events Checklist (LEC)**. The LEC assesses lifetime history of exposure to a wide range of potentially traumatic events.[60] The LEC will be used to describe the study population, and to establish PTSD diagnostic status as part of the inclusion criteria.

Depression. The depression measure at each time point will be from the NIH-sponsored **Patient-Reported Outcomes Measurement Information System (PROMIS)** assessment suite. PROMIS was developed in order to measure patient-reported outcomes using item response theory (IRT) and computerized adaptive testing (CAT) which allows for efficient, psychometrically robust assessment of patient-reported outcomes in clinical trials involving a wide range of chronic conditions, and they have been validated in over 21,000 persons.[10,65] CAT is a method by which the items administered are adaptive to the health status of the subject, i.e., the instrument used is not "static" for each subject. Using CAT and IRT, the PROMIS measures are designed to gain the maximal information content from each question by gauging the point of disease severity a question is most efficient. The greater precision gained by use of these techniques has been estimated to allow reduction in sample size requirements by up to half compared with "static" pen and paper instruments.[65] Most PROMIS item banks utilize a 7-day recall period. These measures utilize item-response theory[66] and computerized adaptive testing (CAT), in order to provide a high degree of precision. Using CAT, each domain measures average 5-8 questions in length and takes only 1-2 minutes to complete. We will utilize the CAT version of the PROMIS depression measure for the major assessment points, and the static version of the PROMIS depression measure (SF8a) at the weekly assessments (using Survey Monkey for electronic data capture).

Self-compassion. The **Self-Compassion Scale – Short Form (SCS-SF)**[61] is a 12-item measure of self-compassion (see also broaden-and-build section of background/significance). It has been shown to be reliable (Cronbach's alpha = 0.86) and to have a very high degree of correlation ($r \geq 0.97$) with the longer and previously validated self-compassion scale.[39]

Posttraumatic Cognitions Inventory (PTCI).[62] The PTCI is a 36-item measure that assesses trauma-related thoughts and beliefs including negative cognitions about self, about the world, and self-blame. Items are rated on a 7-point scale ranging from "totally disagree" to "totally agree." The PTCI has been found to discriminate well between people who do and do not have PTSD and has been found to have good internal consistency, test-retest reliability, and construct validity. The PTCI will be administered at baseline and follow-up time points as a secondary outcome measure. The abbreviated 22-item version[44] will be collected weekly to assess whether changes in posttraumatic cognitions mediate changes in the primary outcome measures.

Weekly monitoring assessment protocol. Weekly assessment of type and duration of LKM and CPT-C homework practices completed along with measures of PTSD and depression and the proposed intervention mediators will be assessed weekly during the twelve weeks of LKM or CPT-C treatment. The weekly assessments will be administered via take home paper assessment. (See Appendix 9 for weekly items.)

Assessment of Usual VA Mental Health Care. In order to quantify the amount of VA mental health care that study participants obtain during their involvement in the proposed study, data extraction from both the VA Corporate Data Warehouse (CDW) and from the computerized patient record system (CPRS). The number of individual and group psychotherapy stopcodes and mental health medication management stopcodes will be recorded for all participants for the period of time they are directly involved in LKM (and equivalent time period for CPT-C) from the CDW. These numbers will be cross-checked via CPRS and CPRS therapy notes will be assessed to determine whether study participants were involved in treatments for PTSD during their involvement in LKM or the follow-up period (and equivalent time period for CPT-C). We will also use CPRS to code for psychiatric and pain medication adjustments during study involvement.

Data Analysis

Overview. Before performing the primary analyses, outliers, shape of distributions, and associations between outcomes will be determined. Covariates or propensity scores will be utilized to adjust for differences if imbalances between treatment groups on key variables are identified. Gender and type of trauma will also be considered as covariates. All analyses will be performed using an intention-to-treat sample as well as per protocol analyses. Per protocol analyses are the preferred method for non-inferiority analyses, in order to

avoid biasing the results toward no difference between groups, which could occur in intention-to-treat samples. In addition, pattern-mixture models will be used to identify potential missing data patterns,[67] and multiple imputation techniques will be used to accommodate missing data.[68]

AIMS 1 and 2. To determine if LKM is therapeutically similar to CPT-C, we will estimate the effect of LKM vs. CPT-C using a multilevel mixed effects model with random effects for repeated measures. If significant correlation exists between outcomes among participants in the same therapy group (i.e., ICC>0), therapy group also will be included as a random effect in the model. The primary outcomes for AIM 1 are participant scores on the CAPS.[60] As the models for AIM 1 and AIM 2 (PTSD and depressive symptoms, respectively) will be identical, we focus on the CAPS. The analysis will include all of the primary assessment time points (Baseline, post-LKM/CPT-C, 3- and 6- months). Mixed models are an appropriate choice for three reasons. First, they accommodate the clustered nature of the data at multiple levels (i.e., repeated measures within persons). Second, should significant correlation exist among participants in the same therapy group, inclusion of group as a random factor allows for generalization of results to LKM and CPT-C groups in general (not just the groups in the study). Third, they are flexible with respect to missing data.[69] Mixed effects models will include treatment condition (LKM vs. CPT-C), time, and time by treatment interaction to determine if differences in exist between conditions by time. Time will be modeled as a dummy variable to allow for variation in effects over time. Both unadjusted and adjusted (covariates include baseline PROMIS depression score for Aim 1 and baseline CAPS for Aim2, age, gender) models will be run. Subgroup effects of PTSD severity on outcomes will be explored using tests of interaction.

A non-inferiority analysis determines if the effects of a new intervention are similar to an established treatment within a pre-stated margin of or range of non-inferiority that is considered clinically acceptable (δ).[70] We chose a non-inferiority margin of 10 points on the CAPS. A difference of 10 points on the CAPS represents Cohen's d = 0.50 in treatment-seeking Vietnam Veterans with PTSD, for whom the SD is roughly 20.[71] A reduction of 10 points on the CAPS is considered the minimal effect that would be clinically meaningful.[71]

Addendum: The CAPS-5 measure became available before the study was initiated and was used in place of the CAPS-IV. We defined a non-inferiority margin on the CAPS-5 using the same methodology as was used for defining the non-inferiority margin using CAPS-IV. The primary outcome of non-inferiority of LKM relative to CPT-C was assessed using a non-inferiority margin of 5 points on the CAPS-5, which represents 0.5 SD of baseline PTSD symptoms based on data indicating the SD of baseline CAPS-5 scores is approximately 10 in a large sample (N=198) of treatment-seeking veterans.[72] An effect size of Cohen's d = 0.5 has been defined as the minimally important difference in prior PTSD trials.[71,73] For depression, the non-inferiority margin was 4 points on the PROMIS depression measure, which has been defined as the minimally important difference and corresponds to a Cohen's d effect size of approximately 0.50.[11,74] A reduction of 4 points on the PROMIS depression measure is the minimal effect considered clinically meaningful, which corresponds to an effect size of approximately 0.5.[11] Hypothesis testing in a non-inferiority analysis is reversed from the standard formulation. The null hypothesis (H_0) states that LKM is at least an amount δ worse than CPT-C. The alternative (H_A) states that the post-treatment mean in LKM is no worse than amount δ as compared to CPT-C.[75] We will assess non-inferiority at 6 month follow-up, as well as other time points (post-LKM/CPT-C, 3-month follow-up) using methods outlined by Mascha and Sessler.[76] Non-inferiority of LKM to CPT-C for reduction in PTSD symptoms will be claimed if the lower limit of the 95% confidence interval for difference in 6-month mean CAPS scores is greater than -10. Non-inferiority of LKM to CPT-C for reduction in depressive symptoms will be claimed if the lower limit of the 95% confidence interval for difference in 6-month mean t-score in the PROMIS depression measure is greater than -4.[11] The test of non-inferiority is similar to the usual test for differences between treatments obtained in a mixed effects model except that the non-inferiority margin δ is added to the treatment effect coefficient b_3 and divided by the standard error.[76]

$$T_{NI} = \frac{b_3 + d}{SE}$$

If non-inferiority is not shown in the above analyses for AIM 1, traditional 'superiority' analyses will be conducted for comparisons between the interventions. This will involve a simple comparison of means at the 6-month time point on the primary outcomes. We chose an effect size of d=.50[51,71] for the comparison of the active treatments. Although the study is powered for the non-inferiority analyses, we also report a power analysis for these preliminary analyses. Assuming an effect of d=.50 between treatment arms and a two-sided α of 0.05 resulted in a power estimate of approximately 90%.

Power and Sample Size:

Sample size for the non-inferiority hypotheses specified in AIM 1 was determined using Stata statistical software.[77] Assuming a non-inferiority margin of 10 points on the CAPS (see rationale noted above) and the typical alpha for a one-sided non-inferiority test (.025),[75] a total sample of 126 participants, (63 PTSD patients per randomization arm) resulted in a power estimate of 80%. *Sample size calculations were unchanged using the CAPS-5 measure as described above.* To protect against the effects of attrition, we added 26% to this final sample size for a recruitment goal of 170 patients. This attrition estimate was based on our pilot study assessing longitudinal outcomes of LKM.

In a preliminary analysis of our LKM pilot data (see preliminary studies section), the intraclass correlation (ICC) for PTSD symptoms (using the PSS-I; n=42) was $\rho = 0.00$ and for depression (using the PROMIS measure) was 0.026 (p = NS for both). Additionally, pilot data based on the VAPSHCS MBSR program showing an ICC for PTSD symptoms (using the PCL; n=118) of $\rho = 0.00$ at post-treatment and $\rho = 0.05$ at follow-up (p = NS). Because our pilot data obtained at our site do not indicate significant correlation of measures within groups, we propose analyses that do not incorporate ICC results and are not powered to account for them.

Exploratory AIM: Mediators of response to LKM and CPT-C

To assess mediation, we will first assess temporality of change of the proposed mediators and outcomes (as described below). If it is established that changes in the proposed mediators precede changes in depressive and PTSD symptoms, we will assess mediation using an analytic framework developed for use in randomized controlled trials of two interventions with equivalent outcomes.[19] The framework developed by Gallagher and Resick[19] (described in more detail below) allows assessment of whether two interventions produce similar outcomes through different mediational processes. In the proposed trial comparing LKM to CPT-C, the two interventions share many common factors, but differ in the focus of the intervention (i.e., LKM emphasizes self-compassion without a focus on cognitive restructuring, whereas CPT-C places a primary emphasis on correcting maladaptive cognitions without a primary focus of developing self-compassion). We propose to examine mechanisms of change for each intervention by assessing intraindividual change in self-compassion and maladaptive cognitions, and then to examine whether intraindividual change in the proposed mediators mediates outcomes (PTSD symptoms, depressive symptoms).

<u>Establishment of Temporal Sequence of Change in Mediators and Outcome Measures</u>: To assess mediation, it is important to assess whether changes in the proposed mediators (self-compassion, posttraumatic maladaptive cognitions) precede change in clinical outcomes (PTSD and depressive symptoms).[78] Weekly measures will be obtained during the treatment phase, in order to provide an assessment schedule that allows measurement of the mediators before improvement occurs in clinical outcomes (PTSD and depressive symptoms). The temporal relationship between the proposed mediators and clinical outcomes will be analyzed according to methods defined by Kleim and colleagues (2012)[44] using bivariate latent growth modeling. Briefly, this first involves modeling weekly latent change processes in mediators and outcomes separately, to find the best representation of latent trajectories. A dual linear growth curve is then calculated, in which latent growth curves are simultaneously estimated for the proposed mediators and the clinical outcomes. Next, the degree to which change in the proposed mediator at week X is associated with change in PTSD at week X+1 (as well as the inverse to test whether changes in depressive/PTSD symptoms predict changes in the proposed mediators) is evaluated to examine whether the requirement of change in the mediator preceding change in the outcome is met. This process assesses whether a larger than average change on one variable is associated with a larger than average change on the other variable at a later time point. Within this framework, when, for example a greater reduction in posttraumatic maladaptive cognitions is associated with a greater reduction in PTSD symptoms in the next session, it is assumed that changes in the first variable cause changes in the second variable. These models will be utilized to determine the optimal time points (from baseline) to assess change scores in mediators and outcomes for the mediation models described below.

<u>Mediation</u>: Within-treatment intraindividual changes will first be analyzed for self-compassion, maladaptive cognitions, PTSD and depression using difference scores (each week's score minus baseline score for each measure) using procedures defined by Gallagher and Resick (2012).[19] The degree to which change in self-compassion mediates change in outcome in LKM relative to CPT-C will be evaluated by examining the indirect effect of treatment condition (LKM vs. CPT-C) on intraindividual change in PTSD symptoms via intraindividual change in self-compassion.

These procedures will be repeated to evaluate the degree to which change in maladaptive cognitions

mediates change in CPT-C relative to LKM by examining the indirect effect of treatment condition (CPT-C vs. LKM) on intraindividual change in PTSD symptoms via intraindividual change in maladaptive cognitions. The above analysis will be repeated for depressive symptoms.

Indirect effects will be calculated to yield bias-corrected and accelerated bootstrapped confidence intervals of indirect effects. If the confidence interval of the indirect effect does not include zero, mediation will be shown.

Project Timeline

Approximately 20-22 Veterans will be recruited for each of the 12 study cohorts. The first cohort will undergo baselines assessments in month 5 (following a 4-month recruitment phase). Cohorts will be approximately evenly distributed over the course of the study. We have previously been successful in obtaining timely regulatory approvals for LKM and MBSR studies at VAPSHCS, including the two randomized pilot trials described in the preliminary studies section.

Limitations

The study design is a fairly ambitious one with a weekly monitoring feature that could pose challenges for retention and compliance with the research protocol. A modest remuneration schedule is likely to help maintain compliance with the study protocol, and our previous research with PTSD and alcohol dependent individuals examining frequent monitoring has shown excellent compliance. Other design considerations include the inclusion of male and female participants with mixed trauma histories and inclusion of individuals with Substance Abuse and/or Alcohol Dependence (AD). Briefly, most PTSD treatment trials have involved fairly homogeneous samples, which while likely reducing experimental noise, also limit the generalizability of the findings. We felt that the ability to obtain data that has a chance of being highly generalizable to Veterans who are seeking care for PTSD, which is more often than not associated with comorbid conditions,[16] was preferable to maintaining strict experimental control. In addition, we have successfully delivered LKM in mixed-gender groups and our clinical impression is that both the male and the female Veterans benefited from the exposure to the opposite gender in this therapeutic context.

Another design consideration is that each of the two interventions will be delivered in closed-group format and there is some risk that people within each of the groups will influence one another to the extent that there are group effects on outcomes.[79] If ICC's are significant in the proposed study, this would result in decreased statistical power to detect the primary outcomes.

Despite limitations the proposed research has considerable conceptual and clinical merit and represents a necessary step in examining whether LKM is associated with clinical benefit for Veterans with PTSD. Results of this research can be readily applied in both VA and community settings to treat PTSD.

Application Summary

The proposed research is an extension of our previous and ongoing work. This application will address a relative void in the intervention literature for PTSD by assessing the non-inferiority of a standardized meditation intervention, LKM. In light of the growing interest in complementary and alternative medicine among Veterans and the lack of empirical data on such approaches for common disorders, such as PTSD, this research will make an important contribution to the field. We believe the proposed research is timely, important, and innovative, and the track records of Drs. Kearney and Simpson and the research team indicate we are capable of successfully carrying out and completing the proposed work.

1. Kessler RC. Posttraumatic stress disorder: the burden to the individual and to society. *Journal of Clinical Psychiatry.* 2000;61:4-12 (suppl 15).
2. Kessler RC, Borges G, Walters EE. Prevalence of and risk factors for lifetime suicide attempts in the national comorbidity survey. *Archives of General Psychiatry.* 1999;56(7):617-626.
3. Davidson JRT. Recognition and treatment of posttraumatic stress disorder. *Jama-Journal of the American Medical Association.* 2001;286(5):584-588.
4. Braet C, Claus L, Goossens L, Moens E, Van Vlierberghe L, Soetens B. Differences in eating style between overweight and normal-weight youngsters. *Journal of Health Psychology.* 2008;13(6):733-743.
5. Wachen JS, Resick P, Mintz, et al. Group Cognitive Processing Therapy for Combat-Related Post-Traumatic Stress Disorder: Main Findings on PTSD and Depression. International Society for Traumatic Stress Studies; November 2, 2012, 2012; Los Angeles, CA.
6. Lombardo TW. Beyond Exposure for Posttraumatic Stress Disorder (PTSD) Symptoms: Broad-Spectrum PTSD Treatment Strategies. *Behavior Modification.* 2005;29(1):3-9.
7. Baldwin CM, Long K, Kroesen K, Brooks AJ, Bell IR. A profile of military veterans in the southwestern United States who use complementary and alternative medicine - Implications for integrated care. *Archives of Internal Medicine.* 2002;162(15):1697-1704.
8. Kroesen K, Baldwin CM, Brooks AJ, Bell IR. US military veterans' perceptions of the conventional medical care system and their use of complementary and alternative medicine. *Family Practice.* 2002;19(1):57-64.
9. Fredrickson BL, Cohn MA, Coffey KA, Pek J, Finkel SM. Open hearts build lives: Positive emotions, induced through loving-kindness meditation, build consequential personal resources. *Journal of Personality and Social Psychology.* 2008;95(5):1045-1062.
10. Cella D, Yount S, Rothrock N, et al. The Patient-Reported Outcomes Measurement Information System (PROMIS) Progress of an NIH roadmap cooperative group during its first two years. *Medical Care.* 2007;45(5):S3-S11.
11. Yost KJ, Eton DT, Garcia SF, Cella D. Minimally important differences were estimated for six Patient-Reported Outcomes Measurement Information System-Cancer scales in advanced-stage cancer patients. *Journal of Clinical Epidemiology.* 2011;64(5):507-516.
12. *American Psychiatric Association [DSM-IV-TR].* Washington, DC2000.
13. Davidson JRT, Stein DJ, Shalev AY, Yehuda R. Posttraumatic stress disorder: acquisition, recognition, course, and treatment. *Journal of Neuropsychiatry & Clinical Neurosciences.* 2004;16(2):135-147.
14. Thomas JL, Wilk JE, Riviere LA, McGurk D, Castro CA, Hoge CW. Prevalence of Mental Health Problems and Functional Impairment Among Active Component and National Guard Soldiers 3 and 12 Months Following Combat in Iraq. *Archives of General Psychiatry.* 2010;67(6):614-623.
15. Eisen SA, Griffith KH, Xian H, et al. Lifetime and 12-month prevalence of psychiatric disorders in 8,169 male Vietnam War era veterans. *Military Medicine.* 2004;169(11):896-902.
16. Kessler RC, Sonnega A, Bromet E, Hughes M, Nelson CB. Posttraumatic-stress-disorder in the national comorbidity survey. *Archives of General Psychiatry.* 1995;52(12):1048-1060.
17. Cougle JR, Resnick H, Kilpatrick DG. PTSD, DEPRESSION, AND THEIR COMORBIDITY IN RELATION TO SUICIDALITY: CROSS-SECTIONAL AND PROSPECTIVE ANALYSES OF A NATIONAL PROBABILITY SAMPLE OF WOMEN. *Depression and Anxiety.* 2009;26(12):1151-1157.
18. Schoenfeld FB, Marmar CR, Neylan TC. Current concepts in pharmacotherapy for posttraumatic stress disorder. *Psychiatric Services.* 2004;55(5):519-531.
19. Gallagher MW, Resick PA. Mechanisms of Change in Cognitive Processing Therapy and Prolonged Exposure Therapy for PTSD: Preliminary Evidence for the Differential Effects of Hopelessness and Habituation. *Cognitive Therapy and Research.* 2012;36(6):750-755.
20. Schnurr PP, Friedman MJ, Engel CC, et al. Cognitive behavioral therapy for posttraumatic stress disorder in women - A randomized controlled trial. *Jama-Journal of the American Medical Association.* 2007;297(8):820-830.
21. Rosen CS, Chow HC, Finney JF, et al. VA practice patterns and practice guidelines for treating posttraumatic stress disorder. *Journal of Traumatic Stress.* 2004;17(3):213-222.
22. Karlin BE, Ruzek JI, Chard KM, et al. Dissemination of Evidence-Based Psychological Treatments for Posttraumatic Stress Disorder in the Veterans Health Administration. *Journal of Traumatic Stress.* 2010;23(6):663-673.

23. Saydah SH, Eberhardt MS. Use of complementary and alternative medicine among adults with chronic diseases: United States 2002. *Journal of Alternative and Complementary Medicine.* 2006;12(8):805-812.
24. Micek MA, Bradley KA, Braddock CH, Maynard C, McDonell M, Fihn SD. Complementary and alternative medicine use among veterans affairs outpatients. *Journal of Alternative and Complementary Medicine.* 2007;13(2):190-193.
25. Neff KD, Rude SS, Kirkpatrick KL. An examination of self-compassion in relation to positive psychological functioning and personality traits. *J Res Pers.* 2007;41(4):908-916.
26. Krupnick JL, Green BL, Stockton P, Miranda J, Krause E, Mete M. Group interpersonal psychotherapy for low-income women with posttraumatic stress disorder. *Psychotherapy Research.* 2008;18(5):497-507.
27. Thompson BL, Waltz J. Self-Compassion and PTSD Symptom Severity. *Journal of Traumatic Stress.* 2008;21(6):556-558.
28. Gilbert P, Procter S. Compassionate mind training for people with high shame and self-criticism: overview and pilot study of a group therapy approach. *Clinical Psychology & Psychotherapy.* 2006;13(6):353-379.
29. Carson JW, Keefe FJ, Lynch TR, et al. Loving-kindness meditation for chronic low back pain. *Journal of Holistic Nursing.* 2005;23(3):287-304.
30. Hutcherson CA, Seppala EM, Gross JJ. Loving-Kindness Meditation Increases Social Connectedness. *Emotion.* 2008;8(5):720-724.
31. Johnson DP, Penn DL, Fredrickson BL, Meyer PS, Kring AM, Brantley M. Loving-kindness meditation to enhance recovery from negative symptoms of schizophrenia. *Journal of Clinical Psychology.* 2009;65(5):499-509.
32. Salzberg S. *Lovingkindness: the revolutionary art of happiness.* Boston, MA: Shambhala Publications, Inc.; 1995.
33. Foa EB, Riggs DS. Posttraumatic stress disorder and rape. *American Psychiatric Press Review of Psychiatry.* 1993;12:273-303.
34. Herman J. *Trauma and Recovery: The Aftermath of Violence--from Domestic Abuse to Political Terror.* Basic Books; 1997.
35. King LA, King DW, Fairbank JA, Keane TM, Adams GA. Resilience-recovery factors in post-traumatic stress disorder among female and male Vietnam veterans: Hardiness, postwar social support, and additional stressful life events. *Journal of Personality and Social Psychology.* 1998;74(2):420-434.
36. Fredrickson BL. The broaden-and-build theory of positive emotions. *Philosophical Transactions of the Royal Society of London Series B-Biological Sciences.* 2004;359(1449):1367-1377.
37. Johnson KJ, Fredrickson BL. "We all look the same to me" - Positive emotions eliminate the own-race bias in face recognition. *Psychological Science.* 2005;16(11):875-881.
38. Rounsaville BJ, Carroll KM, Onken LS. A Stage Model of Behavioral Therapies research: Getting started and moving on from stage I. *Clinical Psychology-Science and Practice.* 2001;8(2):133-142.
39. Neff KD. The development and validation of a scale to measure self-compassion. *Self and Identity.* 2003;2:223-250.
40. Leary MR, Tate EB, Adams CE, Allen AB, Hancock J. Self-compassion and reactions to unpleasant self-relevant events: the implications of treating oneself kindly. *Journal of Personality and Social Psychology.* 2007;92(5):887-904.
41. Kuyken W, Watkins E, Holden E, et al. How does mindfulness-based cognitive therapy work? *Behav Res Ther.* 2010;48(11):1105-1112.
42. Resick PA, Schnicke MK. Cognitive Processing Therapy for Sexual Assault Victims. *Journal of Consulting and Clinical Psychology.* 1992;60(5):748-756.
43. Resick PA, Galovski TE, Uhlmansiek MO, Scher CD, Clum GA, Young-Xu Y. A Randomized clinical trial to dismantle components of cognitive processing therapy for posttraumatic stress disorder in female victims of interpersonal violence. *Journal of Consulting and Clinical Psychology.* 2008;76(2):243-258.
44. Kleim B, Grey N, Wild J, et al. Cognitive Change Predicts Symptom Reduction With Cognitive Therapy for Posttraumatic Stress Disorder. *Journal of Consulting and Clinical Psychology.* 2012;Advance online publication.
45. Longmore RJ, Worrell M. Do we need to challenge thoughts in cognitive behavior therapy? *Clin Psychol Rev.* 2007;27(2):173-187.

46. Tsuang MT, Simpson JC, Koenen KC, Kremen WS, Lyons MJ. Spiritual well-being and health. *Journal of Nervous and Mental Disease.* 2007;195(8):673-680.
47. Litz BT, Gray MJ. Emotional numbing in posttraumatic stress disorder: current and future research directions. *Australian and New Zealand Journal of Psychiatry.* 2002;36(2):198-204.
48. Resick PA, Monson CM, Chard KM. *Cognitive processing therapy: Veteran/military version.* Washington, DC: Department of Veterans Affairs;2008.
49. Resick P. Cognitive therapy for posttraumatic stress disorder. *Journal of cognitive Psychotherapy.* 2001;15(4):321-329.
50. Resick PA, Nishith P, Weaver TL, Astin MC, Feuer CA. A comparison of cognitive-processing therapy with prolonged exposure and a waiting condition for the treatment of chronic posttraumatic stress disorder in female rape victims. *Journal of Consulting and Clinical Psychology.* 2002;70(4):867-879.
51. Monson CM, Schnurr PP, Resick PA, Friedman MJ, Young-Xu Y, Stevens SP. Cognitive processing therapy for veterans with military-related posttraumatic stress disorder. *Journal of Consulting and Clinical Psychology.* 2006;74(5):898-907.
52. Bernardy NC, Hamblen JL, Friedman MJ, Ruzek JI, McFall ME. Implementation of a posttraumatic stress disorder mentoring program to improve treatment services. *Psychological Trauma: Theory, Research, Practice, and Policy.* 2011;3(3):292.
53. Seal KH, Bertenthal D, Miner CR, Sen S, Marmar C. Bringing the war back home - Mental health disorders among 103 788 US veterans returning from Iraq and Afghanistan seen at Department of Veterans Affairs facilities. *Archives of Internal Medicine.* 2007;167(5):476-482.
54. Hollon SD, Munoz RF, Barlow DH, et al. Psychosocial intervention development for the prevention and treatment of depression: Promoting innovation and increasing access. *Biological Psychiatry.* 2002;52(6):610-630.
55. Imel ZE, Wampold BE, Miller SD, Fleming RR. Distinctions Without a Difference: Direct Comparisons of Psychotherapies for Alcohol Use Disorders. *Psychology of Addictive Behaviors.* 2008;22(4):533-543.
56. MacCoon DG, Imel ZE, Rosenkranz MA, et al. The validation of an active control intervention for Mindfulness Based Stress Reduction (MBSR). *Behav Res Ther.* 2012;50(1):3-12.
57. Weathers FW, Keane TM, Davidson JR. Clinician-administered PTSD scale: a review of the first ten years of research. *Depression & Anxiety.* 2001;13(3):132-156.
58. Scott CK, Sonis J, Creamer M, Dennis ML. Maximizing follow-up in longitudinal studies of traumatized populations. *Journal of Traumatic Stress.* 2006;19(6):757-769.
59. Sheehan DV, Lecrubier Y, Sheehan KH, et al. The Mini-International Neuropsychiatric Interview (MINI): The development and validation of a structured diagnostic psychiatric interview for DSM-IV and ICD-10. *Journal of Clinical Psychiatry.* 1998;59:22-33.
60. Blake DD, Weathers FW, Nagy LM, et al. The development of a clinician-administered PTSD scale. *Journal of Traumatic Stress.* 1995;8(1):75-90.
61. Raes F, Pommier E, Neff KD, Van Gucht D. Construction and Factorial Validation of a Short Form of the Self-Compassion Scale. *Clinical Psychology & Psychotherapy.* 2011;18(3):250-255.
62. Foa EB, Ehlers A, Clark DM, Tolin DF, Orsillo SM. The Posttraumatic Cognitions Inventory (PTCI): Development and validation. *Psychological Assessment.* 1999;11(3):303-314.
63. Weathers FW, Litz BT, Herman DS, Huska JA, Keane TM. The PTSD Checklist (PCL): Reliability, validity, and diagnostic utility. Paper presented at the 9th Annual Meeting of the International Society for Traumatic Stress Studies; 1993; San Antonio, TX.
64. Weathers FW, Bovin MJ, Lee DJ, et al. The Clinician-Administered PTSD Scale for DSM-5 (CAPS-5): Development and initial psychometric evaluation in military veterans. *Psychol Assess.* 2018;30(3):383-395.
65. Fries JF, Krishnan E. What constitutes progress in assessing patient outcomes? *Journal of Clinical Epidemiology.* 2009;62(8):779-780.
66. Cook KF, Kallen MA, Amtmann D. Having a fit: impact of number of items and distribution of data on traditional criteria for assessing IRT's unidimensionality assumption. *Quality of Life Research.* 2009;18(4):447-460.
67. Atkins DC. Using multilevel models to analyze couple and family treatment data: Basic and advanced issues. *Journal of Family Psychology.* 2005;19(1):98-110.
68. Little R, Rubin D. *Statistical analysis with missing data.* New York: Wiley; 1987.
69. Raudenbush SW, Bryk AS. *Hierarchichal linear models.* Thousand Oaks, CA: Sage; 2002.

70. Piaggio G, Elbourne DR, Altman DG, Pocock SJ, Evans SJW, Grp C. Reporting of noninferiority and equivalence randomized trials - An extension of the CONSORT Statement. *Jama-Journal of the American Medical Association.* 2006;295(10):1152-1160.
71. Schnurr PP, Friedman MJ, Foy DW, et al. Randomized trial of trauma-focused group therapy for posttraumatic stress disorder - Results from a Department of Veterans Affairs Cooperative Study. *Archives of General Psychiatry.* 2003;60(5):481-489.
72. Marx BP. In:2019.
73. Greene CJ, Morland LA, Durkalski VL, Frueh BC. Noninferiority and Equivalence Designs: Issues and Implications for Mental Health Research. *Journal of Traumatic Stress.* 2008;21(5):433-439.
74. Swanholm E, McDonald W, Makris U, Noe C, Gatchel R. Estimates of Minimally Important Differences (MIDs) for Two Patient-Reported Outcomes Measurement Information System (PROMIS) Computer-Adaptive Tests in Chronic Pain Patients. 2014;19(4):217-232.
75. Laster LL, Johnson MF, Kotler ML. Non-inferiority trials: the 'at least as good as' criterion with dichotomous data. *Statistics in Medicine.* 2006;25(7):1115-1130.
76. Mascha EJ, Sessler DI. Equivalence and Noninferiority Testing in Regression Models and Repeated-Measures Designs. *Anesthesia and Analgesia.* 2011;112(3):678-687.
77. *SSI: Stata module to estimate sample size for randomized controlled trials, Statistical Software Components S457150* [computer program]. Boston College Department of Economics; 2010.
78. Kazdin AE. Mediators and mechanisms of change in psychotherapy research. In: *Annual Review of Clinical Psychology.* Vol 3.2007:1-27.
79. Baldwin SA, Murray DM, Shadish WR. Empirically supported treatments or type I errors? Problems with the analysis of data from group-administered treatments. *Journal of Consulting and Clinical Psychology.* 2005;73(5):924-935.
