# Supplementary material for: Loving-Kindness Meditation vs Cognitive Processing Therapy for Posttraumatic Stress Disorder Among Veterans: A Randomized Clinical Trial
Source: JAMA Netw Open. 2021 Apr 16;4(4):e216604. doi: 10.1001/jamanetworkopen.2021.6604 (PMC8052593; doi:10.1001/jamanetworkopen.2021.6604)
Supplement: Supplement 2. — eAppendix. Additional Description of Loving-Kindness Meditation and Rationale for Its Use as a PTSD Treatment eTable. Within- and Between-Condition Effect Sizes by Treatment Outcome eFigure. Estimated Differences Between Loving-Kindness Meditation and Cognitive Processing Therapy in Outcome Measures by Time, Patients Attending ≥6 Visits eReferences [file jamanetwopen-e216604-s002.pdf]

Supplementary Online Content

Kearney DJ, Malte CA, Storms M, Simpson TL. Loving-kindness meditation vs cognitive processing therapy for posttraumatic stress disorder among veterans: a randomized clinical trial. *JAMA Netw Open*. 2021;4(4):e216604. doi:10.1001/jamanetworkopen.2021.6604

eAppendix. Additional Description of Loving-Kindness Meditation and Rationale for Its Use as a PTSD Treatment

eTable. Within- and Between-Condition Effect Sizes by Treatment Outcome

eFigure. Estimated Differences Between Loving-Kindness Meditation and Cognitive Processing Therapy in Outcome Measures by Time, Patients Attending ≥6 Visits

eReferences.

This supplementary material has been provided by the authors to give readers additional information about their work.

© 2021 Kearney DJ et al. *JAMA Network Open.*

eAppendix. Additional Description of Loving-Kindness Meditation and Rationale for Its Use as a PTSD Treatment

Loving-kindness meditation (LKM) is a practice intended to repeatedly elicit feelings of kindness and compassion for self and others. Although LKM shares some similarities with mindfulness meditation, it is a distinct form of practice. A recent classification considers meditation practices to fall into three broad categories: 1) Attentional meditation practices, which foster self-regulatory skills, 2) Deconstructive meditation practices, which regard thoughts, sensations and emotions as mental events, rather than reflections of reality, and 3) Constructive meditation practices, which strengthen psychological patterns that promote well-being.[1] Within this classification system, the broadly available interventions Mindfulness-Based Stress Reduction and Mindfulness-Based Cognitive Therapy include both attentional and deconstructive elements, whereas LKM is primarily a constructive form of meditation practice.[1] Constructive forms of meditation, such as LKM and compassion meditation, seek to enhance or bolster positive affect and prosocial motivation, whereas mindfulness meditation primarily involves bringing an attitude of non-judgmental attention to the experience of the present moment.

In LKM, development of positive affect and prosocial motivation is achieved through the silent repetition of phrases that invoke the desire for safety, happiness, health, and ease or peace for self or others.[2] A person is asked to inquire into their positive motivations, or intentions, for themselves and others, and to develop personalized phrases that reflect these intentions; these phrases are then repeated silently during periods of meditation. When practicing LKM, individuals are asked to notice any positive affect that is present during the practice, and to regard without judgment and with kindness other aspects of their experience regardless of content - which in the setting of PTSD often includes self-critical or self-limiting thoughts, or unpleasant feelings, images or memories.

Loving-kindness practice has its roots in the Buddhist tradition, but an increasing number of studies have successfully applied it as a non-religious practice.[3-6] The phrase loving-kindness is derived from the Pali word *metta*, [2,3] which can be translated as “love” or “unconditional friendliness,” or “loving-kindness,” akin to the Greek word “agape,” which is typically translated as wide open, unconditional love. The words loving-kindness are intended to describe an emotional state that is not a sentimental love or a feeling of passion. Rather, it can be described as an unconditional friendliness, benevolence, and openness toward experience – even difficult experience.[2] Mechanistically, the process of enhancing kind thoughts and wishes for others, as is taught in LKM, is theorized to generate *de novo* positive affect and motivational states, which involves activation of brain areas distinct from those involved in cognitive reappraisal strategies.[7] Functional neuroimaging studies indicate that the positive affect

© 2021 Kearney DJ et al. *JAMA Network Open.*

elicited by loving-kindness practice occurs in parallel with negative emotion elicited when a person confronts and feels one's suffering or the suffering or others.[7]

In the setting of PTSD, the generation of positive affect by LKM is theorized to increase the ability to tolerate, rather than avoid or suppress, distressing thoughts, images, and feelings, and to counteract shame, and guilt - central symptoms of PTSD.[8,9] Ultimately, an increased ability to engage distressing states is theorized to facilitate exposure, which over time results in diminished reactivity to stressors through the process of habituation.[10] Many people with chronic PTSD also experience a diminished ability to feel positive emotions, especially those involving intimacy or affection, which can result in feelings of chronic alienation and emotional deadness. By providing a technique through which positive emotions are repeatedly elicited, LKM practice might, in theory, provide an innovative pathway to address symptoms of emotional numbing.[11] Furthermore, the repetition of LKM phrases could, in theory, provide a technique to provide self-soothing, which would replace habitual patterns of self-critical thinking. The ability to self-soothe might be particularly helpful to people with PTSD when kindness and support are lacking in the environment, given that social support is an important factor in recovery from trauma.[12-14]

There is a small but growing literature evaluating LKM interventions in the setting of PTSD. A pilot randomized controlled trial (N=28) of veterans with PTSD compared Cognitively-Based Compassion Training® to a combined psychoeducation/relaxation/sleep hygiene control condition, both delivered in group format, and found greater PTSD symptom reduction for those assigned to the compassion intervention.[15] An RCT compared loving-kindness and compassion meditation with a non-specified control condition for female trauma survivors, all of whom had substance use disorders (N = 63), and showed improvement in trauma symptoms over time for only the LKM group as well as large between-group effects.[16] In addition, a before-and-after pilot study of group-based LKM for military veterans with PTSD (N = 42) showed significant improvement in PTSD symptoms, depression and positive affect, as well as reductions in negative affect over time.[8,9] Although not a trial of LKM or self-compassion, an additional study of patients with PTSD sheds light on the possible mechanistic role of changes in self-compassion as they relate to changes in PTSD. Specifically, a temporal sequencing study involving residential care patients with PTSD who were undergoing exposure therapy (N = 67) assessed whether self-compassion mediated change in PTSD and lagged models demonstrated that elevated self-compassion was associated with significantly decreased PTSD severity three days later.[17]

<u>Additional Description of the LKM format used in the study.</u> The LKM curriculum is based on the

© 2021 Kearney DJ et al. *JAMA Network Open.*

formulation described by Salzberg.[2] In LKM, one calls to mind a particular person (e.g., a good friend) and silently repeats phrases that invoke goodwill, such as: “may you be safe,” “may you be happy,” and “may you be healthy.” Over 12 weeks, the practice gradually expands to bring to mind other individuals or categories of people, including oneself, a benefactor, neutral persons, those who have caused difficulty or harm, and all beings - changing the phrases as needed (i.e., “May you be safe” becomes “May I be safe”).[2] Animals, such as a trusted pet, can also be incorporated into practice. While practicing LKM, a person is asked to notice and feel any positive emotions that arise after repeating the phrases of positive intention, or to notice if there is a sense of reluctance, hesitation, or even aversion for oneself or others. Participants are asked to notice thoughts and feelings elicited by the phrases with an attitude of kindness, curiosity, and non-judgment, regardless of content. During LKM, when a person becomes distracted by thoughts that arise during the practice, they are instructed to notice the distraction, then without judgment, return to the LKM phrases. While practicing LKM, a person is also encouraged to remain grounded in the feeling of the breath and the body. Walking meditation is also used as a method to teach LKM; the pace of walking is synchronized with repetition of LKM phrases. Class sessions began with a brief mindfulness (weeks 1 and 2) or loving-kindness (weeks 3 through 12) meditation followed by discussion and additional LKM practice. Outside of class, participants are encouraged to practice LKM in informal settings such as while eating, washing the dishes, or waiting in line at the grocery store. Educational materials describing the relationship between meditation, PTSD, and depression are provided. Homework consists of 30 minutes of meditation 6 days per week using audio guides in addition to the informal LKM practices in daily life.

© 2021 Kearney DJ et al. *JAMA Network Open.*

eTable. Within- and Between-Condition Effect Sizes by Treatment Outcome

	Baseline to End of Treatment	Baseline to 3-Mo Follow-up	Baseline to 6-Mo Follow-up
**ITT**			
CAPS (n = 183)*			
Within-Condition Effects			
CPT	0.47 (0.18, 0.76)	0.48 (0.18, 0.77)	0.52 (0.22, 0.81)
LKM	0.47 (0.18, 0.77)	0.54 (0.24, 0.84)	0.66 (0.36, 0.96)
Between-Condition Effects	0.01 (-0.28, 0.30)	0.07 (-0.22, 0.36)	0.13 (-0.16, 0.42)
PROMIS Depression T-Score			
Within-Condition Effects			
CPT	-0.11 (-0.40, 0.18)	-0.21 (-0.50, 0.08)	-0.09 (-0.38, 0.19)
LKM	0.39 (0.09, 0.68)	0.28 (-0.01, 0.57)	0.28 (-0.01, 0.58)
Between-Condition Effects	0.35 (0.06, 0.64)	0.34 (0.05, 0.63)	0.24 (-0.05, 0.53)
**Participants Completing ≥6 Treatment Sessions (N=111)**			
CAPS			
Within-Condition Effects			
CPT	0.54 (0.14, 0.94)	0.52 (0.12, 0.92)	0.46 (0.07, 0.86)
LKM	0.45 (0.09, 0.81)	0.49 (0.13, 0.85)	0.64 (0.28, 1.01)
Between-Condition Effects	-0.14 (-0.51, 0.23)	-0.06 (-0.44, 0.31)	0.13 (-0.25, 0.50)
PROMIS Depression T-Score			
Within-Condition Effects			
CPT	-0.18 (-0.58, 0.21)	-0.23 (-0.62, 0.16)	-0.12 (-0.51, 0.27)
LKM	0.40 (0.04, 0.76)	0.24 (-0.12, 0.59)	0.38 (0.02, 0.73)
Between-Condition Effects	0.54 (0.16, 0.92)	0.41 (0.03, 0.79)	0.42 (0.04, 0.80)

Note: Data from one veteran is missing from the PTSD models because the subject did not complete the PROMIS depression at baseline, which is a covariate.

© 2021 Kearney DJ et al. *JAMA Network Open.*

eFigure. Estimated Differences Between Loving-Kindness Meditation and Cognitive Processing Therapy in Outcome Measures by Time, Patients Attending ≥6 Visits

eFigure 1. Estimated Differences Between Loving-Kindness Meditation and Cognitive Processing Therapy in Outcome Measures by Time, Patients Attending ≥6 Visits

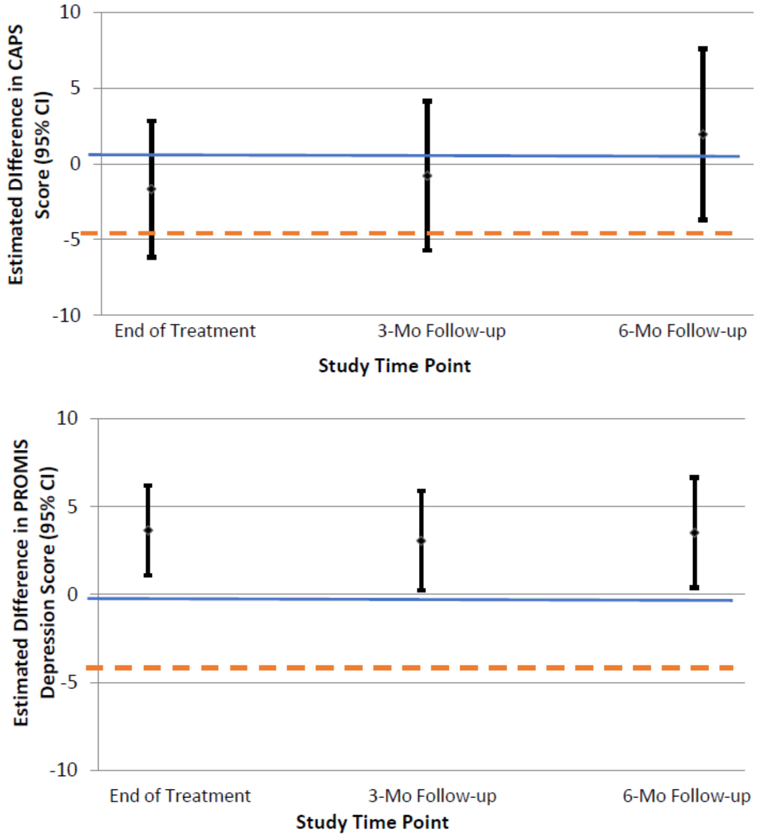

© 2021 Kearney DJ et al. *JAMA Network Open.*

eReferences

1. Dahl CJ, Lutz A, Davidson RJ. Reconstructing and deconstructing the self: cognitive mechanisms in meditation practice. *Trends Cogn Sci.* 2015;19(9):515-523.
2. Salzberg S. *Lovingkindness: the revolutionary art of happiness.* Boston, MA: Shambhala Publications, Inc.; 1995.
3. Graser J, Stangier U. Compassion and Loving-Kindness Meditation: An Overview and Prospects for the Application in Clinical Samples. *Harv Rev Psychiatry.* 2018;26(4):201-215.
4. Galante J, Galante I, Bekkers MJ, Gallacher J. Effect of kindness-based meditation on health and well-being: a systematic review and meta-analysis. *J Consult Clin Psychol.* 2014;82(6):1101-1114.
5. Hofmann SG, Grossman P, Hinton DE. Loving-kindness and compassion meditation: potential for psychological interventions. *Clin Psychol Rev.* 2011;31(7):1126-1132.
6. Fredrickson BL, Cohn MA, Coffey KA, Pek J, Finkel SM. Open hearts build lives: Positive emotions, induced through loving-kindness meditation, build consequential personal resources. *Journal of Personality and Social Psychology.* 2008;95(5):1045-1062.
7. Engen HG, Singer T. Affect and Motivation Are Critical in Constructive Meditation. *Trends Cogn Sci.* 2016;20(3):159-160.
8. Kearney DJ, McManus C, Malte CA, Martinez ME, Felleman B, Simpson TL. Loving-Kindness Meditation and the Broaden-and-Build Theory of Positive Emotions Among Veterans With Posttraumatic Stress Disorder. *Medical Care.* 2014;52:S32-S38.
9. Kearney DJ, Malte CA, McManus C, Martinez M, Felleman B, Simpson TL. Loving-Kindness Meditation for Posttraumatic Stress Disorder: A Pilot Study. *Journal of Traumatic Stress.* 2013;26(4):426-434.
10. Foa EB, Kozak MJ. Emotional processing of fear: exposure to corrective information. *Psychological Bulletin.* 1986;99(1):20-35.
11. Kearney DJ, Simpson TL. *Mindfulness-based interventions for trauma and its consequences.* Washington, DC: American Psychological Association; 2020.
12. King LA, King DW, Fairbank JA, Keane TM, Adams GA. Resilience-recovery factors in post-traumatic stress disorder among female and male Vietnam veterans: Hardiness, postwar social support, and additional stressful life events. *Journal of Personality and Social Psychology.* 1998;74(2):420-434.
13. Heron-Delaney M, Kenardy J, Charlton E, Matsuoka Y. A systematic review of predictors of posttraumatic stress disorder (PTSD) for adult road traffic crash survivors. *Injury.* 2013;44(11):1413-1422.
14. Brewin CR, Andrews B, Valentine JD. Meta-analysis of risk factors for posttraumatic stress disorder in trauma-exposed adults. *J Consult Clin Psychol.* 2000;68(5):748-766.
15. Lang AJ, Malaktaris AL, Casmar P, et al. Compassion Meditation for Posttraumatic Stress Disorder in Veterans: A Randomized Proof of Concept Study. *J Trauma Stress.* 2019;32(2):299-309.
16. Lee MY, Zaharlick A, Akers D. Impact of Meditation on Mental Health Outcomes of Female Trauma Survivors of Interpersonal Violence With Co-Occurring Disorders: A Randomized Controlled Trial. *J Interpers Violence.* 2017;32(14):2139-2165.
17. Hoffart A, Oktedalen T, Langkaas TF. Self-compassion influences PTSD symptoms in the process of change in trauma-focused cognitive-behavioral therapies: a study of within-person processes. *Frontiers in psychology.* 2015;6:1273.

© 2021 Kearney DJ et al. *JAMA Network Open.*
