# Supplementary material for: Loving-Kindness Meditation vs Cognitive Processing Therapy for Posttraumatic Stress Disorder Among Veterans: A Randomized Clinical Trial
Source: JAMA Netw Open. 2021 Apr 16;4(4):e216604. doi: 10.1001/jamanetworkopen.2021.6604 (PMC8052593; doi:10.1001/jamanetworkopen.2021.6604)
Supplement: Supplement 3. — Data Sharing Statement [file jamanetwopen-e216604-s003.pdf]

Data Sharing Statement

Kearney. Loving-Kindness Meditation vs Cognitive Processing Therapy for Posttraumatic Stress Disorder Among Veterans. *JAMA Netw Open*. Published April 16, 2021. doi:10.1001/jamanetworkopen.2021.6604

Data
Data available: No

Additional Information
Explanation for why data not available: The consent process performed at the VA did not include obtaining permission from participants to make the data available. Given that our informed consent process did not include the provision that we would share the data, we are unable to share individual patient data.
